# A Phase I Randomized Therapeutic MVA-B Vaccination Improves the Magnitude and Quality of the T Cell Immune Responses in HIV-1-Infected Subjects on HAART

**DOI:** 10.1371/journal.pone.0141456

**Published:** 2015-11-06

**Authors:** Carmen Elena Gómez, Beatriz Perdiguero, Juan García-Arriaza, Victoria Cepeda, Carlos Óscar Sánchez-Sorzano, Beatriz Mothe, José Luis Jiménez, María Ángeles Muñoz-Fernández, Jose M. Gatell, Juan Carlos López Bernaldo de Quirós, Christian Brander, Felipe García, Mariano Esteban

**Affiliations:** 1 Department of Molecular and Cellular Biology, Centro Nacional de Biotecnología, Consejo Superior de Investigaciones Científicas (CSIC), Madrid, Spain; 2 IrsiCaixa-HIVACAT, Hospital Universitari Germans Trias i Pujol, Autonomous University of Barcelona, Badalona, Spain; 3 Hospital Universitario Gregorio Marañón, Madrid, Spain; 4 Hospital Clinic-HIVACAT, IDIBAPS, Barcelona, Spain; 5 Institució Catalana de Recerca i Estudis Avancats (ICREA), Barcelona, and University of Vic and Central Catalonia, Vic, Spain; Rush University, UNITED STATES

## Abstract

**Trial Design:**

Previous studies suggested that poxvirus-based vaccines might be instrumental in the therapeutic HIV field. A phase I clinical trial was conducted in HIV-1-infected patients on highly active antiretroviral therapy (HAART), with CD4 T cell counts above 450 cells/mm^3^ and undetectable viremia. Thirty participants were randomized (2:1) to receive either 3 intramuscular injections of MVA-B vaccine (coding for clade B HIV-1 Env, Gag, Pol and Nef antigens) or placebo, followed by interruption of HAART.

**Methods:**

The magnitude, breadth, quality and phenotype of the HIV-1-specific T cell response were assayed by intracellular cytokine staining (ICS) in 22 volunteers pre- and post-vaccination.

**Results:**

MVA-B vaccine induced newly detected HIV-1-specific CD4 T cell responses and expanded pre-existing responses (mostly against Gag, Pol and Nef antigens) that were high in magnitude, broadly directed and showed an enhanced polyfunctionality with a T effector memory (TEM) phenotype, while maintaining the magnitude and quality of the pre-existing HIV-1-specific CD8 T cell responses. In addition, vaccination also triggered preferential CD8^+^ T cell polyfunctional responses to the MVA vector antigens that increase in magnitude after two and three booster doses.

**Conclusion:**

MVA-B vaccination represents a feasible strategy to improve T cell responses in individuals with pre-existing HIV-1-specific immunity.

**Trial Registration:**

ClinicalTrials.gov NCT01571466

## Introduction

Highly active antiretroviral therapy (HAART) has dramatically improved the clinical outcome in human immunodeficiency virus (HIV)-infected individuals through sustained suppression of viral replication [1]. However, HAART alone is unable to clear HIV-1 infection, likely in part due to the persistence of viral reservoirs [2–5]. Despite the benefits associated with HAART use, long-term drug regimens fail for many patients due to drug resistance, non-adherence or toxicity [6]. Therefore, there is an urgent need to develop therapeutic strategies that can eliminate persistent viral reservoirs and boost host immunity to control HIV-1 viral replication upon discontinuation of HAART. Therapeutic HIV-1 vaccination is one approach that could potentially achieve these goals through the stimulation of effective HIV-1-specific T cell responses; primarily cytolytic/virus suppressive CD8 T cells with supporting CD4 T help [7–11].

Attenuated poxviruses such as canarypox, fowlpox, NYVAC and Modified Vaccinia Virus Ankara (MVA) have been applied in several therapeutic HIV-1 vaccination trials with encouraging results in terms of activation of HIV-1-specific T cell responses, but the outcomes of viral load reduction after HAART interruption have been quite discrepant (for review [11]).

We have previously shown that an MVA vector expressing the HIV-1 Env, Gag, Pol and Nef antigens from clade B (termed MVA-B) was safe and broadly immunogenic when tested in a phase I clinical trial in human healthy volunteers, inducing broad, polyfunctional and long-lasting HIV-1-specific CD4 and CD8 T cell responses, as well as humoral responses against Env in most of the vaccinees [12, 13]. Moreover, vector replication and expression of HIV-1 antigens by MVA-B were not affected when HIV-1 protease inhibitors were used during *in vitro* infection [14].

We have recently described a therapeutic HIV-1 vaccination phase I clinical trial (termed RISVAC03), consisting of 3 doses of MVA-B vaccination, and reported to be safe, well tolerated and with the result of increasing Gag-specific T cell responses when analyzed by ELISPOT assay. A delay in viral rebound in patients was observed but was not associated with immunogenicity and vaccination did not have an impact in viral reservoir even when the vaccine was given in combination with disulfiram [15]. To extend these findings, here we have evaluated by intracellular cytokine staining (ICS) the magnitude, breadth, polyfunctionality and phenotypic profile of the HIV-1-specific CD4 and CD8 T cellular immune responses elicited in chronically HIV-1-infected subjects on HAART, before and after MVA-B administration. We have also assessed the ability of this therapeutic approach to induce MVA vector-specific T cell responses following booster doses of the vaccine. Our findings showed the CD4 and CD8 T cell immunological impact of MVA-B vaccination in HIV-1-infected individuals on HAART.

## Materials and Methods

### Ethics Statement

The RISVAC03 study was approved by the Hospital Clinic (Barcelona), Hospital Germans Trias i Pujol (Badalona) and Hospital Gregorio Marañón (Madrid) ethical review boards (Authorized: June 2, 2011) and by the Spanish Regulatory Authorities (Clinical Trials.gov identifier: NCT01571466; Registered: November 9, 2011). The study was explained to all patients in detail and all participants signed written informed consent documents. The authors confirm that all ongoing and related trials for this study are registered. The protocol of the RISVAC03 clinical trial is detailed in S1 Protocol and the checklist with specific information related with this study can be consulted in S1 CONSORT Checklist.

### Study design

**Fig 1:**
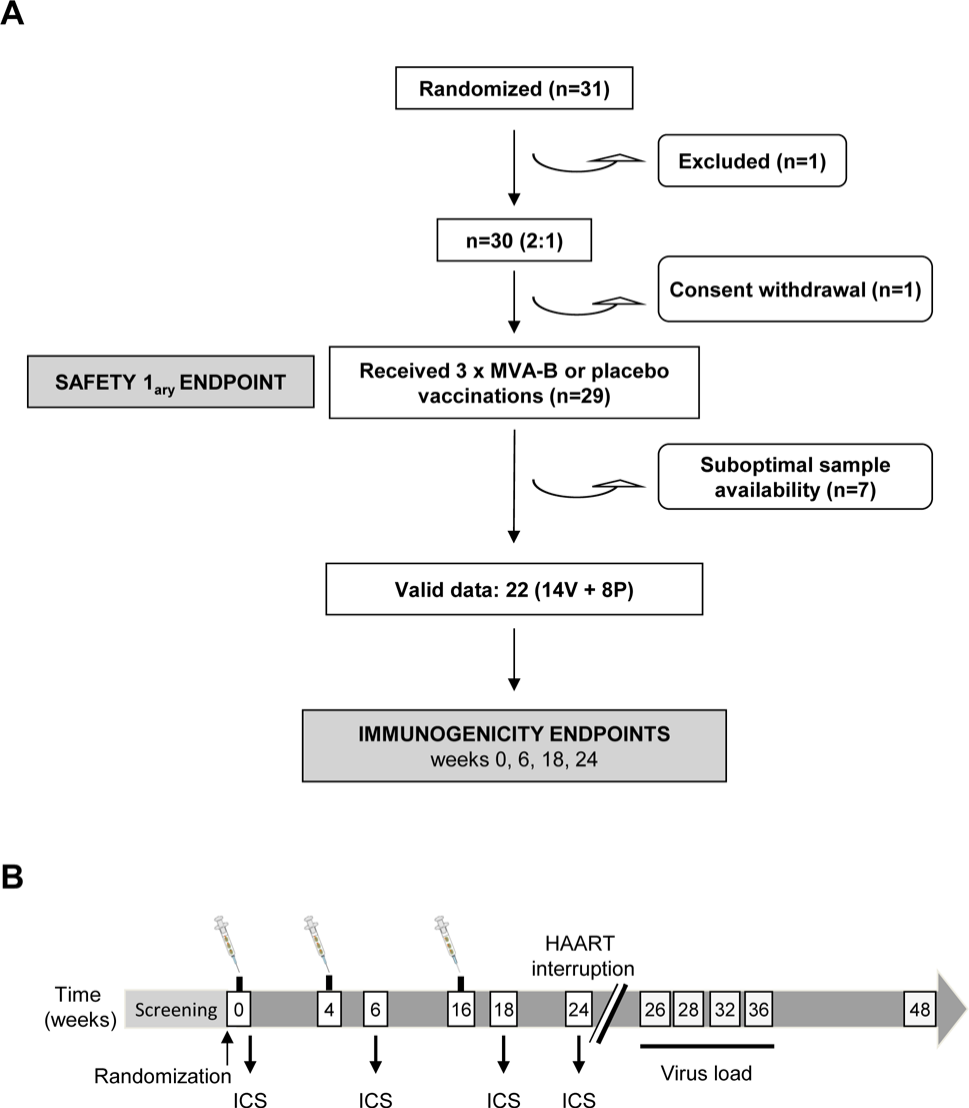
Flow chart of the RISVAC03 study design and distribution of volunteers. (A) A total of 31 volunteers were randomized (21 to the MVA-B arm and 10 to the placebo arm) but one was excluded. One volunteer withdrew consent and 29 volunteers received three doses of the vaccine or placebo. Seven patients had suboptimal sample availability so the immunogenicity to vaccination or placebo was assessed by ICS in 22 volunteers. All 29 participants were included in the safety analysis. V: vaccine; P: placebo. (B) Chronological diagram showing the vaccination schedule, the HAART interruption timeline, the immunogenicity endpoints and the determination of HIV-1 viral loads.

A total of 30 chronic HIV-1-infected patients on HAART with CD4 T cell counts above 450 cells/mm^3^ and undetectable viremia (<50 copies/ml) were enrolled in a phase I, double-blind, placebo-controlled trial conducted at three HIV-1 units in Barcelona (Hospital Clinic), Badalona (Hospital Germans Trias i Pujol) and Madrid (Hospital Gregorio Marañón), Spain, between November 2011 and December 2013. The random allocation and blinding of volunteers were defined at each clinical center and the clinical characteristics of the patients have been previously described [15]. One of the volunteers withdrew his consent to the study. For the phase I therapeutic clinical trial, the volunteers were randomly allocated to receive three 0.5 ml injections of MVA-B (10^8^ PFU/dose) (n = 20) or placebo (n = 10) by intramuscular route at weeks 0, 4 and 16. HAART was interrupted 8 weeks after the last dose of MVA-B (week 24). The safety profile, immunogenicity evaluated by ELISPOT and HIV-1 plasma viral load (pVL) rebound after HAART interruption of the RISVAC03 clinical trial have been recently described [15]. Cryopreserved peripheral blood mononuclear cells (PBMCs) were provided by the Spanish HIV HGM BioBank (RED RIS). Blood samples were processed following current procedures and frozen immediately after reception [16]. For the present study, sufficient cell material was available to assess the antigen-specific T cell immune responses in 22 HIV-1-infected patients (14 vaccinees and 8 placebos). Note that the dropout reasons (withdrawal of consent or lack of sufficient biological material for the analysis) are unrelated to the treatment, so that the losses can be considered to be random and no bias is expected by the lack of these samples. PBMCs were analyzed by polychromatic ICS assay from pre- (week 0) and post-vaccination (weeks 6, 18 and 24) samples. The duration of participant follow-up was 48 weeks (Fig 1). A table showing baseline demographic and clinical characteristics of each group has been previously reported [15].

### MVA-B vaccine

MVA-B vector was derived from the attenuated MVA poxvirus strain and its generation has previously been described [17]. It expresses simultaneously and under the same synthetic early/late viral promoter the genes encoding for monomeric gp120 from the HIV-1 primary isolate BX08 as a cell-released product and Gag-Pol-Nef (GPN) from HIV-1 clone IIIB as an intracellular polyprotein of 160 kDa. MVA-B was genetically stable, even when grown and purified at a large scale under good manufacturing practices (GMP) as previously described [17].

### HIV-1 peptides

All HIV-1 peptides used were >80% purity and provided by EuroVacc Foundation. Overlapping peptides (15-mers with 11 amino acids overlapping, n = 450) spanning the entire HIV-1 Env, Gag, Pol and Nef regions from clade B included in the MVA-B vector were grouped in eight pools and the amino acid position and number of peptides per pool have been previously described [13]. For immunological analyses they were clustered as follows: Env pool (Env-1 and Env-2), Gag pool (Gag-1 and Gag-2) and GPN pool (GPN-1, GPN-2, GPN-3 and GPN-4). The Gag-derived peptides included in the Gag pool are not repeated in the GPN pool.

### Flow cytometric assays

Cryopreserved PBMCs were thawed and rested overnight in RPMI 1640 medium containing 10% FBS. After resting, PBMCs were stimulated during 6 h in complete RPMI 1640 medium containing 1 μl/ml GolgiPlug (BD Biosciences), 2 μM monensin (eBioscience), anti-CD107a-FITC (BD Biosciences) and 1 ug/ml each of the different HIV-1 peptide pools. For the detection of anti-vaccinia response, PBMCs were stimulated as described above but using as stimulus autologous PBMCs infected with MVA at 2 PFU/cell in a ratio of 2:1. At the end of the stimulation period, cells were washed, stained for the surface markers, fixed and permeabilized (Cytofix/Cytoperm Kit; BD Biosciences) and stained intracellularly using the appropriate fluorochromes. Dead cells were excluded using the violet LIVE/DEAD stain kit (Invitrogen). For functional analyses, the following fluorochrome-conjugated antibodies were used: CD4-Alexa 700, CD8-APC-H7, IFN-γ-PerCP-Cy5.5, IL-2-APC and TNF-α-PE-Cy7. In addition, for phenotypic analyses, the following antibodies were used: CCR7-PE and CD45RA-PE-CF594. All antibodies were from BD Biosciences. Cells were acquired using a GALLIOS flow cytometer (Beckman Coulter). Analyses of the data were performed using FlowJo software version 8.5.3 (Tree Star, Ashland, OR). The average number of total events acquired was about 3 x 10^5^ cells. After gating, boolean combinations of single functional gates were then created using FlowJo software to determine the frequency of each response based on all possible combinations of cytokines expression or all possible combinations of differentiation markers expression. Background responses detected in negative control tubes (non-stimulated PBMCs) were subtracted from those detected in stimulated samples for every specific functional combination.

### Data analysis and statistics

In order to compare the different immune responses to the different stimuli, we computed for each patient and each time point the proportion, p_1_, of cells responding to the specific stimuli. We, then, computed the probability distribution associated to this experimental measurement as a Beta probability density function with N_responding_+1 and N_total_-N_responding_+1 degrees of freedom [18, 19] (being N_responding_ the number of responding cells and N_total_ the total number of cells). In our procedure, we estimate the a posteriori probability density function of the estimated proportion. The confidence intervals of our estimates do not need to be symmetric and they are not based on any assumption (other than the proportion is between 0 and 1). In those comparisons in which two time points were involved (t_0_ and t_F_), we calculated for each individual the probability density function of the random variable Δp = p_F_-p_0_ (i.e., we explicitly considered the paired nature of samples) by calculating the integral implied by the subtraction (note that the probability density distributions of p_F_ and p_0_ are known thanks to a previously published methodology [19]). Then, we used a Wilcoxon signed rank test to check whether there was a difference between the p_1_ populations of two time points or two groups.

Analysis and presentation of distributions were performed using MATLAB 2013 and SPICE version 5.1, downloaded from http://exon.niaid.nih.gov [20]. Comparison of distributions was performed using a Student's T test and a partial permutation test as described [20]. All values used for analyzing proportionate representation of responses are background-subtracted.

## Results

### MVA-B vaccination significantly improves the frequency, magnitude and breadth of HIV-1-specific CD4 T cell responses, while maintaining HIV-1-specific CD8 T cell responses

Vaccine-induced T cell responses were assessed in 22 volunteers (14 vaccinees and 8 placebos) by ICS assay after the stimulation of PBMCs with the different HIV-1 peptide pools (Env, Gag and GPN). The HIV-1-specific CD4 T cell responses were based on the frequency of IFN-γ and/or TNF-α and/or IL-2-producing cells, whereas the CD8 T cell responses were based on the frequency of CD107a and/or IFN-γ and/or TNF-α and/or IL-2-producing cells. For each T cell subset, the response was considered positive if the value in the stimulated sample was 3-fold the value obtained in the non-stimulated control and if the background-subtracted magnitude was higher than 0.02%. The background for the different cytokines in the non-stimulated controls never exceeded 0.015%.

**Fig 2:**
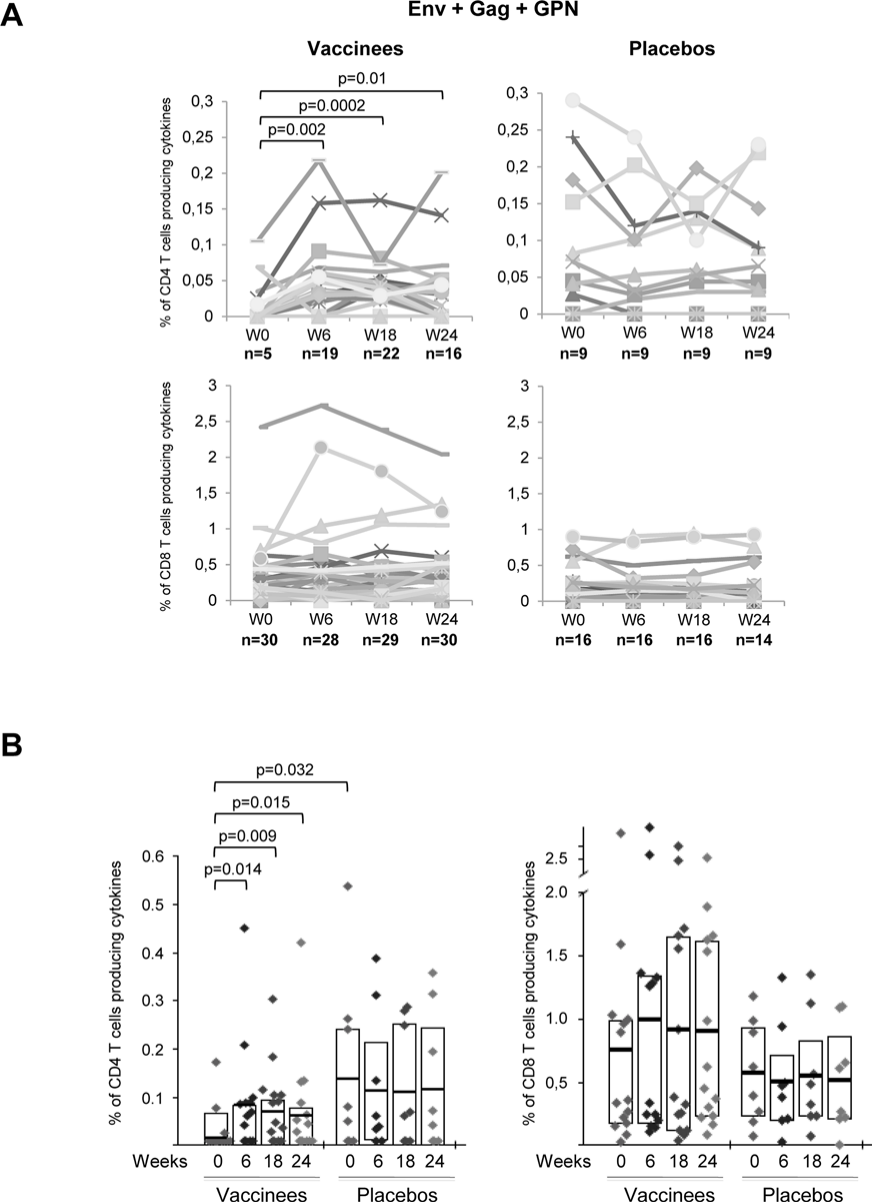
Overall HIV-1-specific T cell responses induced throughout the study. (A) Total HIV-1-specific (Env+Gag+GPN) CD4 and CD8 T cell responses in vaccinated and placebo groups before vaccination (W0), after two (W6) or three (W18) MVA-B or placebo doses or at time of HAART interruption (W24). n values represent the number of positive Env-, Gag- and GPN-specific T cell responses at each time point. *p*-values for significant differences in the proportion of responses were determined using the χ^2^ test for the equality of proportions. (B) Magnitude of the total HIV-1-specific (Env+Gag+GPN) CD4 and CD8 T cell responses in vaccinees and placebos pre- and post-vaccination. The boxes indicate the mean and interquartile range (IQR). *p*-values for significant differences were determined using the Wilcoxon rank sum test with continuity correction and are represented. All data are background-subtracted.

As shown in Fig 2A in vaccinees, the analysis of the number of positive Env-, Gag- and GPN-specific CD4 T cell responses revealed that MVA-B immunization significantly induced newly detected HIV-1 antigen-specific CD4 T cell responses after two (W6, n = 19 total responses, p = 0.002) or three (W18, n = 22, p = 0.0002) doses compared to baseline time point (W0, n = 5). CD4 T cell responses generated persisted at the time of HAART interruption (W24, n = 16, p = 0.01) relative to last vaccination. However, the number of positive Env-, Gag- and GPN-specific CD8 T cell responses did not show any significant temporal variation associated with the administration of the vaccine. On the other hand, HIV-1-specific CD4 and CD8 T cell responses did not change over time in placebo group (Fig 2A). Increases in the magnitude of the pre-existing HIV-1-specific CD4 and CD8 T cell responses were also detected after MVA-B immunization in 64.3% and 50% of the vaccinees, respectively, although statistical significances were only achieved for CD4^+^ T cells (all p<0.05) (Fig 2B). Before vaccination, the magnitude and frequency of HIV-1-specific CD4 T cell responses in placebo group were significantly higher than in vaccinees (p = 0.032) but these responses were maintained throughout all the time points analyzed, while in MVA-B recipients we observed the expansion and appearance of newly detected HIV-specific CD4 T cell responses over time indicating that re-exposure to the HIV antigens expressed by MVA-B vector has a benefit on the overall immune responses (Fig 2B).

**Fig 3:**
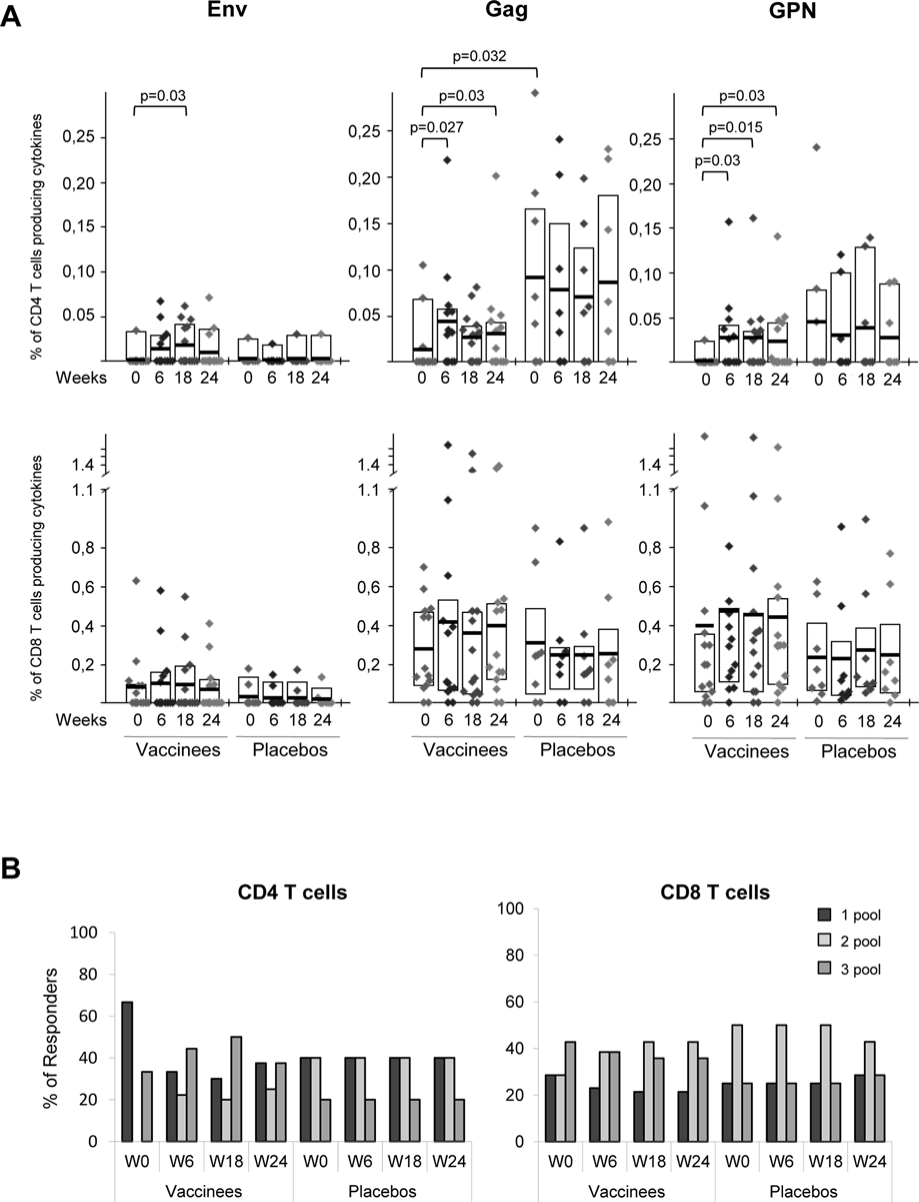
Frequency and breadth of HIV-1-specific CD4 and CD8 T cell responses. (A) Frequency of CD4 and CD8 T cell responses against Env, Gag or GPN peptide pools in vaccinated and placebo groups before vaccination (W0), after two (W6) or three (W18) MVA-B or placebo doses or at time of HAART interruption (W24). The boxes indicate the mean and IQR. *p*-values for significant differences were determined using the Wilcoxon rank sum test with continuity correction and are represented. All data are background-subtracted. (B) Breadth of HIV-1-specific CD4 and CD8 T cell responses at the different time points. Percentages of vaccinees and placebos that recognize 1, 2 or 3 HIV-1 peptide pools in both T cell subsets are shown.

**Fig 4:**
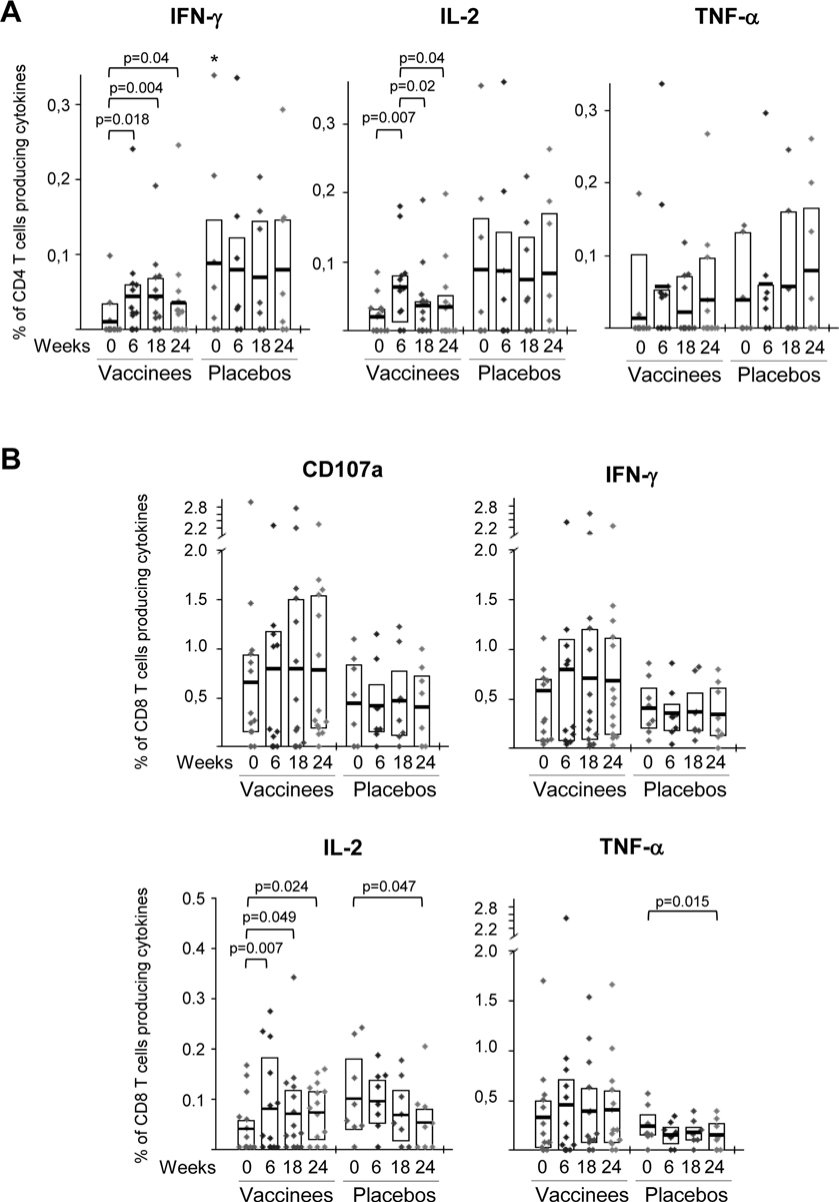
Percentages of HIV-1-specific CD4 and CD8 T cells secreting cytokines or mobilizing CD107a. Frequency of HIV-1-specific CD4 (A) and CD8 (B) T cells producing IFN-γ, TNF-α, IL-2 or mobilizing CD107a. The box plots show the distribution of responses in vaccinees and placebos before vaccination (W0), after two (W6) or three (W18) MVA-B or placebo doses or at time of HAART interruption (W24). The boxes indicate the mean and IQR. Data points represent the sum of the frequencies obtained against Env, Gag and GPN peptide pools. *p*-values were determined using the Wilcoxon rank sum test with continuity correction and are represented. Asterisk indicates that significant differences between vaccinated and placebo groups were observed at this time point. All data are background-subtracted.

This increase of the overall HIV-1-specific CD4 T cell responses detected post-vaccination in vaccinees was mostly due to an enhancement in the frequency and magnitude of the Gag- and GPN-specific responses and to a lesser extent due to Env-specific responses (Fig 3A, upper panels). In the case of the newly detected HIV-1-specific CD4 T cells, we could not distinguish if they were derived from naïve CD4 T cells or from pre-existing HIV-1-experienced CD4 T cell responses which were below the threshold level of detection. HIV-1-specific CD8 T cell responses were detected in all of the 22 volunteers pre- and post-vaccination, with preference in the order of Gag = GPN>Env. Although MVA-B vaccination induced an increase in the magnitude of the Gag- and GPN-specific CD8 T cell responses, these differences were not significant (Fig 3A, lower panels). In the placebo group, the HIV-1-specific CD4 and CD8 T cell responses pre- and post-vaccination were mainly directed against Gag and GPN, with the Gag-specific CD4 T cell responses before vaccination being significantly higher than the response detected in subjects receiving the vaccine (p = 0.032) (Fig 3A). As shown in Fig 3B, in the vaccinated group most (67%) individuals showed pre-existing HIV-1-specific CD4 T cell responses to a single peptide pool only, while after MVA-B immunization there was an increase in the breadth, with more than 60% of the vaccinees reacting against 2 or 3 peptide pools (Fig 3B, left panel). In contrast, the HIV-1-specific CD8 T cell responses were broad and evenly distributed to 1, 2 or 3 HIV-1 peptide pools at all time points assayed (Fig 3B, right panel). In the placebo group, the breadth of the CD4 and CD8 HIV-1-specific T cell responses remained unchanged before and after vaccination (Fig 3B). In terms of total cytokine production, MVA-B vaccination induced a significant increase in the percentages of HIV-1-specific CD4 T cells secreting IFN-γ or IL-2 (Fig 4A). This was also the case for CD8 T cells secreting IL-2 (Fig 4B), since the secretion of the other cytokines or the surface mobilization of CD107a in CD8 T cells were not significantly affected by the vaccine (Fig 4B).

Overall these data demonstrated that MVA-B vaccine significantly improved the magnitude and breadth of the pre-existing HIV-1-specific CD4 T cell responses, while maintaining the HIV-1-specific CD8 T cell responses.

### MVA-B vaccination significantly improved the polyfunctionality and T effector memory phenotype of pre-existing HIV-1-specific CD4 T cell responses, while maintaining the strength of HIV-1-specific CD8 T cell responses

**Fig 5:**
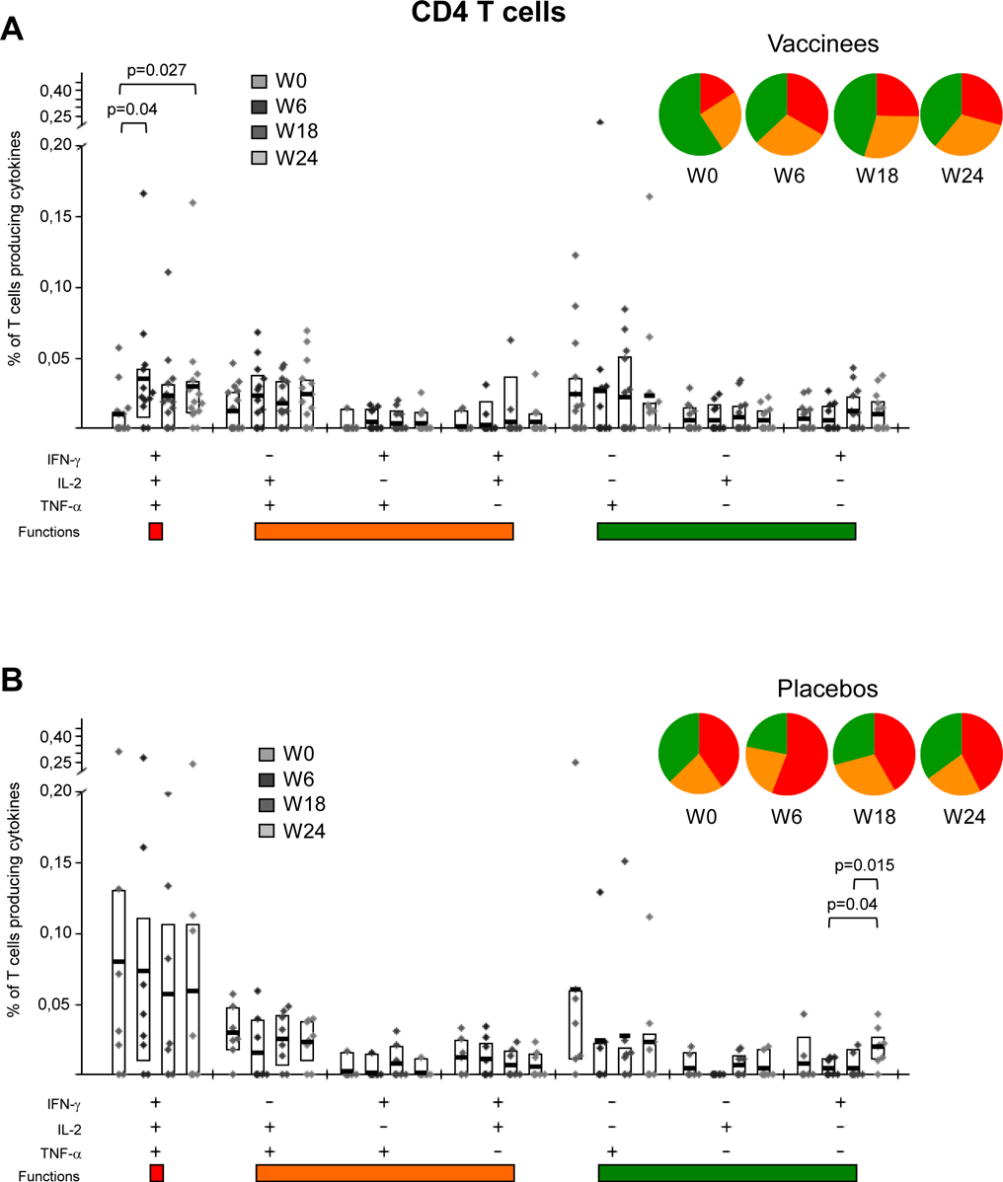
Functional profile of HIV-1-specific CD4 T cells. Frequencies of HIV-1-specific CD4 T cells (directed against Env+Gag+GPN) that express IFN-γ and/or IL-2 and/or TNF-α in vaccinated (A) or placebo (B) groups before vaccination (W0), after two (W6) or three (W18) MVA-B or placebo doses or at time of HAART interruption (W24). All the combinations of the different functions contributing to the overall HIV-1-specific responses are shown on the *x* axis, whereas the percentages of the functionally distinct cell populations within cytokine-producing CD4^+^ T cells are shown on the *y* axis. Responses are grouped and color-coded on the basis of the number of functions. The boxes correspond to the individual data points and IQR at the different time points. The pie charts show the average proportion of the HIV-1-specific CD4 T cell responses according to the functions. Comparison of distributions was performed using a Student's T test and a partial permutation test as described [20]. All data are background-subtracted.

**Fig 6:**
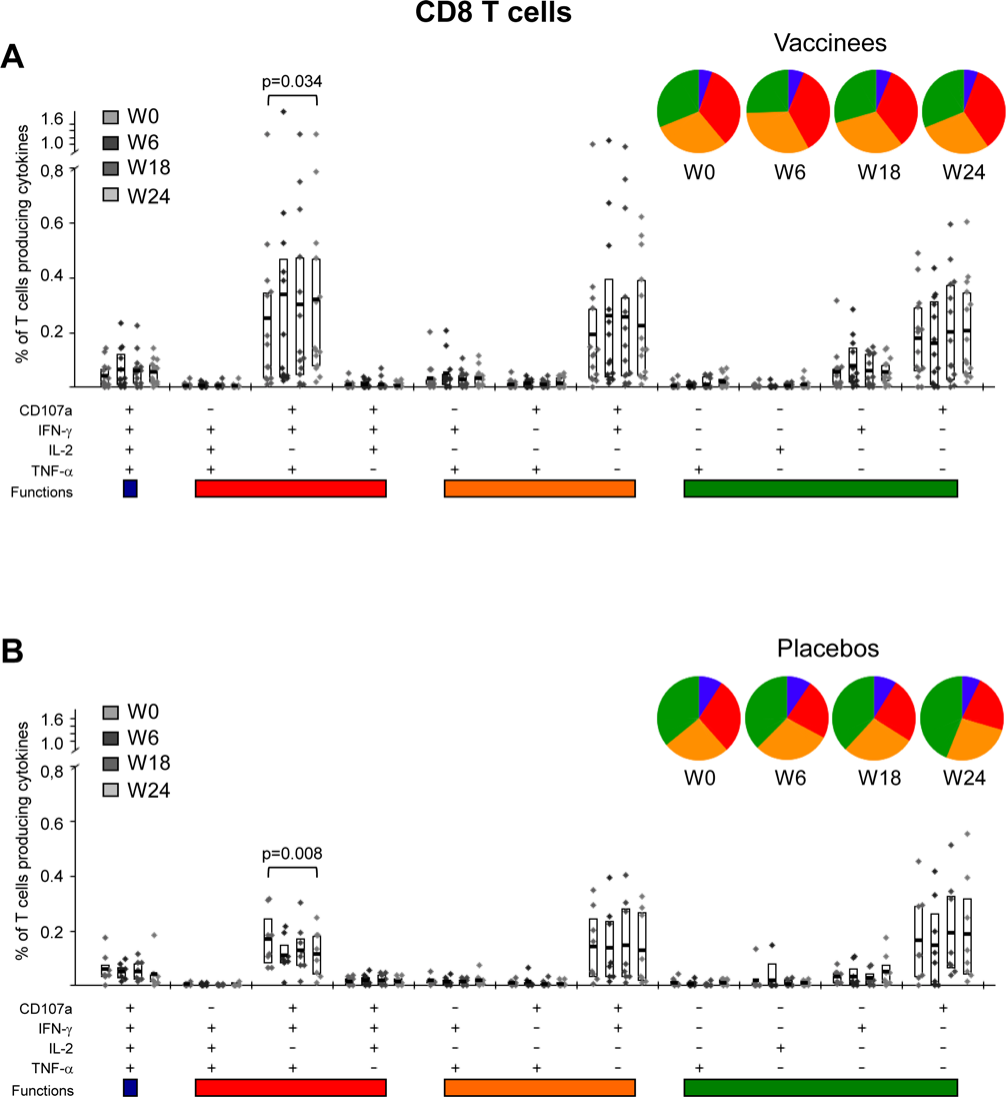
Functional profile of HIV-1-specific CD8 T cells. Frequencies of HIV-1-specific CD8 T cells (directed against Env+Gag+GPN) that mobilize CD107a and/or express IFN-γ and/or IL-2 and/or TNF-α in vaccinated (A) or placebo (B) groups before vaccination (W0), after two (W6) or three (W18) MVA-B or placebo doses or at time of HAART interruption (W24). All the combinations of the different functions contributing to the overall HIV-1-specific responses are shown on the *x* axis, whereas the percentages of the functionally distinct cell populations within cytokine-producing CD8^+^ T cells are shown on the *y* axis. Responses are grouped and color-coded on the basis of the number of functions. The boxes correspond to the individual data points and IQR at the different time points. The pie charts show the average proportion of the HIV-1-specific CD8 T cell responses according to the functions. Comparison of distributions was performed using a Student's T test and a partial permutation test as described [20]. All data are background-subtracted.

The functional and phenotypic profiles of HIV-1-specific CD4 and CD8 T cell responses were analyzed pre- and post-vaccination by polychromatic ICS assay. For functional analyses, we quantified the intracellular production of IFN-γ, IL-2 and TNF-α by HIV-1 Env-, Gag- and GPN-specific CD4 T cells and additionally evaluated the surface mobilization of CD107a by MVA-B immunogen-specific CD8 T cells. Pre-existing HIV-1-specific T cell responses were highly polyfunctional in both vaccinee and placebo groups, with more than 40% of the CD4 T cells and 60% of the CD8 T cells in the vaccinee group exhibiting at least two effector functions (Figs 5 and 6). Still, MVA-B immunization improved the functional profile of the HIV-1-specific CD4 T cells by further increasing the frequencies of T cells secreting simultaneously IFN-γ+IL-2+TNF-α (Fig 5A). Furthermore, MVA-B vaccination induced an increase in the frequencies of HIV-1-specific CD8 T cells secreting simultaneously CD107a+IFN-γ+TNF-α (Fig 6A). In the placebo arm, the functional profile of the HIV-1-specific CD4 and CD8 T cells was the same at pre- and post-vaccination times (Figs 5B and 6B).

**Fig 7:**
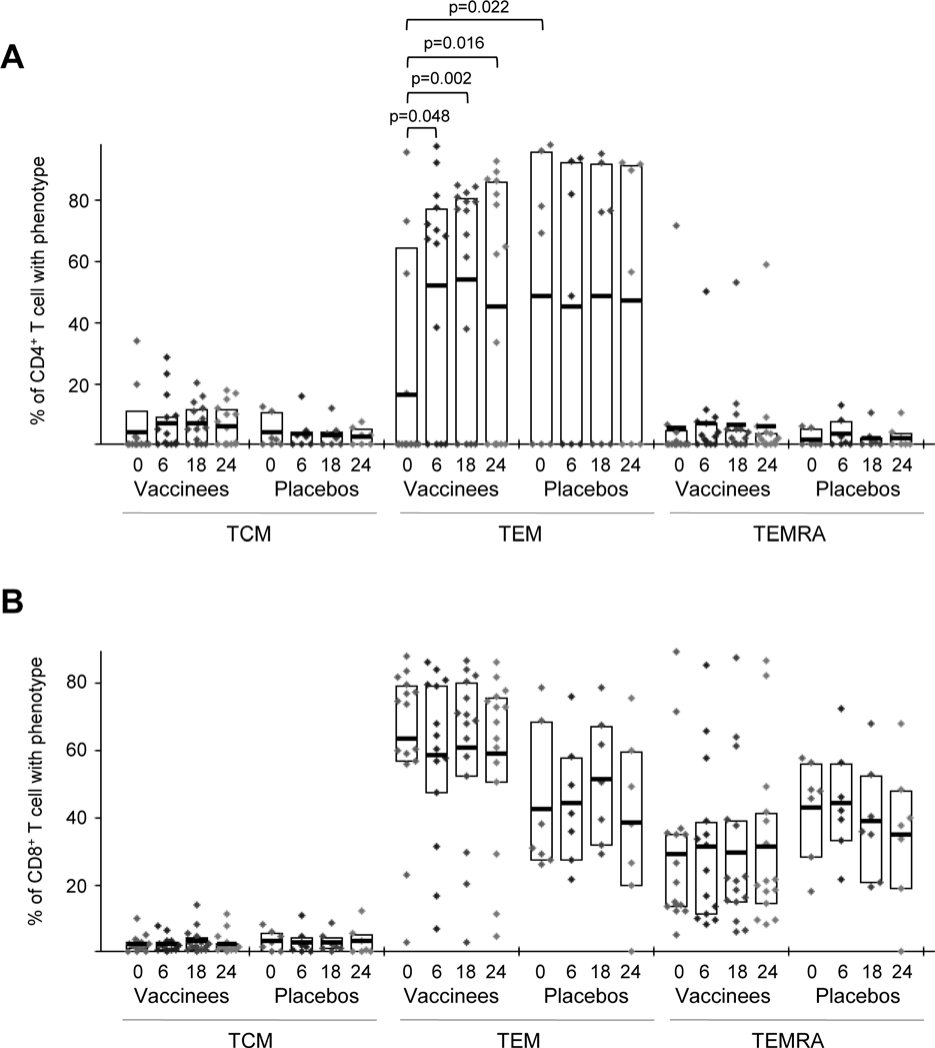
Phenotype of the HIV-1-specific CD4 and CD8 T cell responses. Frequencies of HIV-1-specific CD4 (A) and CD8 T cells (B) (directed against Env+Gag+GPN) with phenotype T central memory (TCM: CD45RA^-^ CCR7^+^), T effector memory (TEM: CD45RA^-^ CCR7^-^) or terminally differentiated T effector memory (TEMRA: CD45RA^+^ CCR7^-^), in vaccinees and placebos before vaccination (W0), after two (W6) or three (W18) MVA-B or placebo doses or at time of HAART interruption (W24). The boxes correspond to the individual data points and IQR at the different time points. *p*-values for significant differences were determined using the Wilcoxon rank sum test with continuity correction and are represented. All data are background-subtracted.

We also characterized the differentiation stages of the responding HIV-1-specific CD4 and CD8 T cells into T central memory (TCM: CD45RA^-^ CCR7^+^), T effector memory (TEM: CD45RA^-^ CCR7^-^) or terminally differentiated T effector memory (TEMRA: CD45RA^+^ CCR7^-^) populations as previously described [21]. HIV-1-specific CD4 T cell responses were mostly of TEM phenotype prior to immunization in both vaccinees and placebos and the percentage of CD4^+^ T cells with TEM phenotype in placebo group was significantly higher than in vaccines (p = 0.022) but only in MVA-B recipients the proportion of this population significantly enhanced (about 3-fold) over time (Fig 7A). Furthermore, the pre-existing HIV-1-specific CD8 T cell responses were mostly of TEM phenotype and to a lesser extent of TEMRA phenotype in both the vaccinee and placebo arms but were unchanged post-vaccination (Fig 7B).

### MVA-B vaccination induced anti-vector specific T cell responses

**Fig 8:**
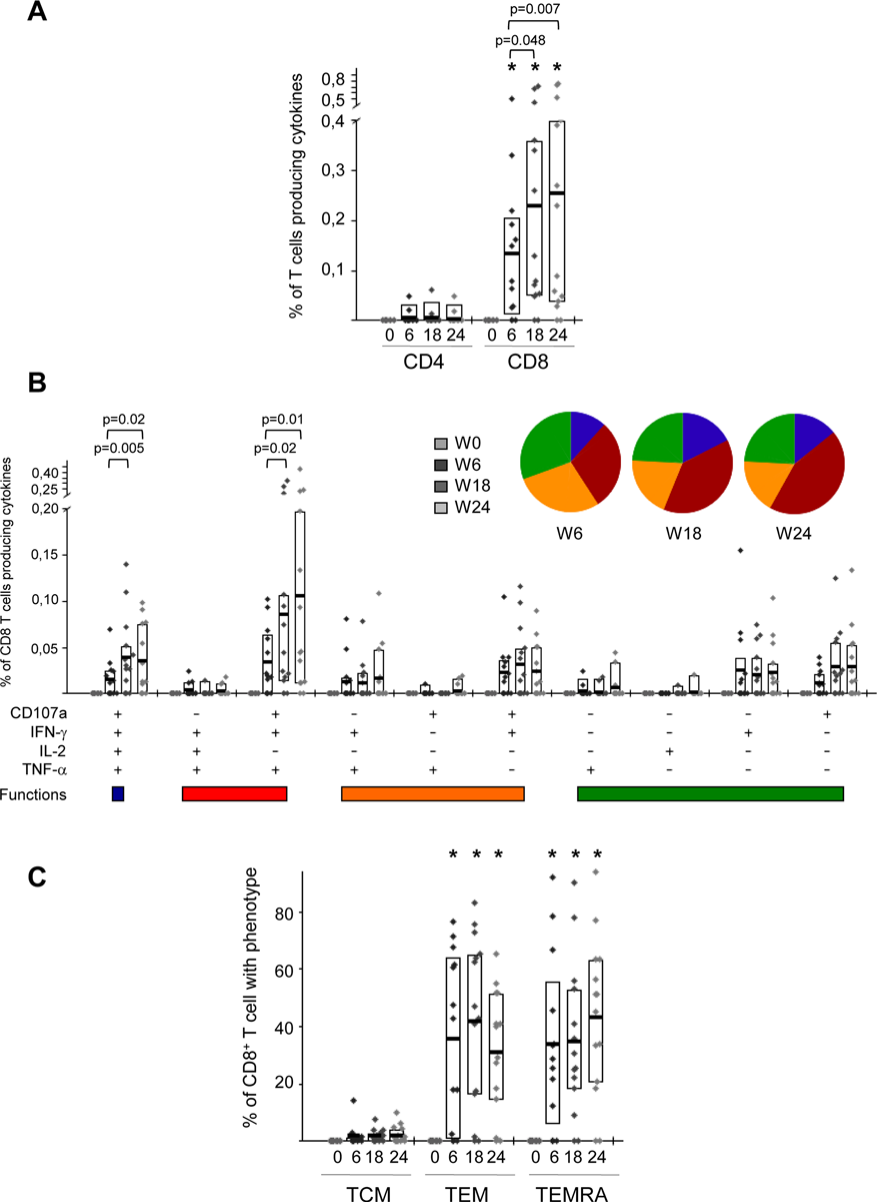
Anti-vector-induced T cell responses throughout the study. (A) Percentages of CD4 and CD8 T cell responses against MVA-infected cells at the different time points in the vaccinee group. The boxes indicate the mean and interquartile range (IQR). *p*-values for significant differences were determined using the Wilcoxon rank sum test with continuity correction and are represented. Asterisks indicate that significant differences between vaccinated and placebo groups were observed at the different time points. All data are background-subtracted. (B) Frequencies of MVA-specific CD8 T cells that mobilize CD107a and/or express IFN-γ and/or IL-2 and/or TNF-α in vaccinated subjects. All the combinations of the different functions contributing to the overall MVA-specific responses are shown on the *x* axis, whereas the percentages of the functionally distinct cell populations within cytokine-producing CD8^+^ T cells are shown on the *y* axis. Responses are grouped and color-coded on the basis of the number of functions. The pie charts show the average proportion of the MVA-specific CD8 T cell responses according to the functions. Comparison of distributions was performed using a Student's T test and a partial permutation test as described [20]. All data are background-subtracted. (C) Frequencies of MVA-specific CD8 T cells with phenotype central memory (TCM: CD45RA^-^ CCR7^+^), T effector memory (TEM: CD45RA^-^ CCR7^-^) or terminally differentiated T effector memory (TEMRA: CD45RA^+^ CCR7^-^) in vaccinated subjects. The boxes correspond to the individual data points and IQR at the different time points. *p*-values for significant differences were determined using the Wilcoxon rank sum test with continuity correction and are represented. Asterisks indicate that significant differences between vaccinated and placebo groups were observed at the different time points. All data are background-subtracted.

We next wanted to define to what extent vaccinees responded to the antigens brought by the MVA vector along different boosters. Anti-vector specific T cell responses were assessed by ICS assay after the stimulation of isolated PBMCs with autologous cells infected with vaccinia virus (VACV) strain MVA. This approach gives higher and more functional T cells than stimulation with peptide-pulsed PBMCs. None of the placebo recipients had positive T cell responses against the vector pre- and post-vaccination. In the vaccine group, no anti-VACV CD8 T cell responses were detected at pre-vaccination time point (W0) but were significantly boosted after 2 or 3 doses of MVA-B vaccine (Fig 8A), with anti-vector specific T cell responses detected in 12 out of 14 (86%) vaccinees. These vaccine-induced anti-vector responses were highly polyfunctional, with about 70% of MVA-specific CD8 T cells displaying more than one function (Fig 8B) and of TEM or TEMRA phenotype (Fig 8C). There was no correlation between the vector specific immunity and the magnitude or breadth of the HIV-1-specific T cell responses.

These findings showed that HIV-infected individuals under HAART treatment responded to the MVA-B vaccine, by triggering specific polyfunctional CD8^+^ T cell responses to the vector that were enhanced after two and three vaccine boosters. This vector-specific immunity does not interfere with the magnitude or breadth of the HIV-1-specific T cell responses elicited, highlighting the advantage of using poxvirus-based vectors as vaccine candidates.

## Discussion

The desired clinical outcome of a therapeutic HIV-1 vaccine is to keep the viral load under control and restore CD4 T cell counts through CD8 T cell-mediated suppression of viral replication. Many different approaches have been assayed to achieve these results. They included non-antigen specific (e.g. using cytokines such as IL-2, IL-12, IFN-γ, IL-7, IL-15, IL-21 or blocking negative regulatory receptors such as PD-1 or CTLA-4) and HIV-1 antigen-specific immune therapies using diverse platforms such as DNA vectors, viral vectors or dendritic cell-based therapeutic vaccines [7–11]. More recently, immunotherapy based on broadly neutralizing antibodies has been shown to reduce viremia in HIV-infected individuals, offering a new modality for HIV prevention, therapy and cure [22]. However, none of the therapeutic studies have been able to control HIV infection for long periods of time, indicating that combined approaches with vaccines and/or antivirals could be a strategy to enhance protective efficacy.

In this study we have evaluated the T cell immunological profile of a poxvirus-based vaccine expressing the HIV-1 Env, Gag, Pol and Nef antigens from clade B (MVA-B) in HIV-1-infected individuals on HAART, through detailed ICS analysis of CD4 and CD8 T cell responses to both HIV-1 and MVA vector antigens. We have recently reported that this therapeutic HIV-1 vaccination protocol was safe and well tolerated and increased Gag-specific T cell responses, when analyzed by ELISPOT assay [15]. Furthermore, we have previously demonstrated that the MVA-B vaccine was safe and broadly immunogenic when tested in a phase I clinical trial in human healthy volunteers [12, 13].

ICS analysis of the HIV-1-specific T cell immune responses elicited by MVA-B vaccination in HIV-1-infected patients on HAART revealed the expansion and also the appearance of newly detected HIV-1-specific CD4 T cell responses, mostly against Gag and GPN HIV-1 antigens, that were high in magnitude, broad, polyfunctional and of TEM phenotype. Moreover, MVA-B also improved, but not significantly (p>0.05), the magnitude of pre-existing HIV-1-specific CD8 T cell responses preserving their antigen-specificity, frequency, breadth, polyfunctionality and phenotypic profile.

The immunological profile of the immune responses induced by MVA-B vaccination observed in this investigation was associated with functional characteristics of T cell responses that have been linked with an effective control of HIV-1 virus replication and with protection against disease progression such as: preferential targeting of Gag epitopes (Fig 3A), concomitant CD4^+^ and CD8^+^ T cell responses (Figs 2, 3 and 7), higher capacity to secrete multiple cytokines and chemokines (polyfunctionality) (Figs 4, 5 and 6), particularly IL-2, or higher expression of lytic factors such as perforin, granzymes and CD107a expression (Fig 4) [23–28]. The fact that the MVA-B vaccine stimulated CD4^+^ T cells with no adverse effects on the patients was an added advantage, as CD4 T cells are the targets of HIV infection and maintenance of these cells favors a better health status of the patients. The role of CD4^+^ T cells in maintaining HIV-specific cellular and humoral responses has long been supported by previous and recent studies, highlighting the potential therapeutic benefits this could have in relation to either HIV remission or eradication [29, 30]. Hence, the boost in HIV-specific CD4 T cell responses that we have observed in our therapeutic phase I clinical trial with MVA-B may contribute to reduce HIV viral load and help on its eradication.

Previously, different MVA-based therapeutic candidates have been evaluated in HIV-1-infected subjects on HAART [31]. They expressed different HIV-1 antigens such as Nef [32, 33], clade A p24/17 and multiple CD8 T cell epitopes [34–36], Gag [37] or multiple antigens (Env, Tat, Rev, Nef and RT) [38]. These candidates were shown to be immunogenic to different extents, inducing both CD4 and CD8 antigen-specific T cell responses with a polyfunctional profile. However, these past studies did not evaluate how the improvements in the immune responses correlated with virological control after treatment interruption. In addition, a NYVAC-based vaccine candidate (termed NYVAC-B) expressing the same HIV-1 antigens as our MVA-B candidate, induced broad and polyfunctional HIV-1-specific T cell responses; however, the antiviral effects after vaccination were not evaluated either [39]. Similarly to MVA-B, NYVAC-B induced in HIV-1-infected patients on HAART the appearance of newly detected HIV-1-specific CD4 T cell responses and an increase in Gag-specific T cell responses [39]. Previous studies have also assayed the potential of poxvirus-derived candidates based on ALVAC or fowlpox strains [40–43]. In most of them, the efficacy in HIV-1 viral control was determined after discontinuation of HAART but only in those protocols that combined the vaccine with cytokines such as IL-2 or IFN-γ a partial prevention of viral rebound was observed. The protection was correlated with the presence of HIV-1-specific CD4 T cell responses [40, 41], IgG2 antibodies to HIV p24 [42, 43] and anti-V1/V2 antibodies [42].

In the RISVAC03 study we have previously observed that MVA-B vaccination induced a modest delay in viral rebound after HAART interruption in a subset of vaccinees [15]. Although plasma viral load (pVL) rebounded in all patients, within a subset of vaccinees we could distinguish between subjects with different dynamics of pVL rebound considering the mean value of pVL at different times after HAART interruption or the time at which they reached 50% of pre-HAART pVLs as indicators of virus control. This delay might be related with the increased polyfunctional CD4^+^ T cell response and TEM phenotype observed in our analysis. Due to the limited availability of PBMCs we could not perform a complete correlation study in order to assess how CD4 and CD8 T cell immunity affected viral rebound. However, we conducted a sub-study of HIV-1 viral rebound dynamics in 9 subjects (6 vaccinees and 3 placebos) during the first 12 weeks after HAART interruption. Although no correlation was obtained due to the low number of individuals, we observed that at the time when HAART was interrupted (W24) the vaccinees with a dynamic of delayed pVL rebound have higher frequencies of Gag-specific CD8 T cells, higher percentages of HIV-1-specific CD4 T cells with TCM phenotype and higher titers of anti-V1/V2 gp120 binding antibodies. However, these immune parameters were not sufficient to provide HIV-1 viral control in the vaccinees. Hence, it seems rather necessary to improve other immunological features which have been previously defined to be associated with a better virus control and prevention of disease progression [26, 28, 44]. These improvements could be done by heterologous prime/boost protocols [45] in combination with reactivating strategies using potent substances that could promote the eradication of HIV-1 in latently infected cells (for review [46]). Alternatively, the combined effects of MVA-B vaccine enhancing T cell responses, together with infusion of broadly neutralizing antibodies might favor a more effective control of HIV infection. Undoubtedly, therapeutic clinical trials are needed to provide detailed information on immune parameters associated with virus control.

In conclusion, our study demonstrates that MVA-B is a broadly immunogenic HIV/AIDS vaccine candidate that stimulated polyfunctional HIV-specific CD4 T cell responses with a T effector memory (TEM) phenotype in HIV-1-infected patients on HAART, and also enhances vector-specific CD8 T cell responses. Thus, HIV-1 immunotherapy using MVA-B in combination with other strategies is a viable option to be explored in future clinical trials.

## Supporting Information

S1 CONSORT Checklist Checklist of RISVAC03 clinical trial. (DOC)

S1 Protocol RISVAC03 clinical trial study protocol. (PDF)
