# Supplementary material for: A Phase I Randomized Therapeutic MVA-B Vaccination Improves the Magnitude and Quality of the T Cell Immune Responses in HIV-1-Infected Subjects on HAART
Source: PLoS One. 2015 Nov 6;10(11):e0141456. doi: 10.1371/journal.pone.0141456 (PMC4636254; doi:10.1371/journal.pone.0141456)
Supplement: S1 Protocol — (PDF) [file pone.0141456.s002.pdf]

RisVac 03
A double-blind phase I study to evaluate the safety of the HIV-1 vaccine MVA-B in chronic HIV-1 infected patients successfully treated with HAART

The trial will be conducted at Hospital Clinic-HIVACAT
Principal Investigator Dr. Felipe García fgarcia@clinic.ub.es
00 34 932275586

Infectious Diseases Unit
Hospital Clínic
Villarroel, 170
08036 Barcelona, Spain Fax: 00 34 934514438

at Hospital Germans Trias i Pujol/IRSICAIXA-HIVACAT
Principal Investigator Dr. Bonaventura Clotet BClotet@irsicaixa.es
00 34 934978849

HIV Unit
Hospital Germans Trial i Pujol
Ctra. de Canyet, s/n
08915 Badalona, Spain Fax: 00 34 934657602

and at the Hospital Gregorio Marañón
Principal Investigator Dr. Juan Carlos López Bernaldo de Quirós
jclopez@mx3.redestb.es
00 34 915868565

Infectious Diseases Unit
Hospital Gregorio Marañón
Dr, Esquerdo, 46
28007 Madrid, Spain Fax: 00 34 915866621

The trial will be coordinated by the Hospital Clinic Clinical Trials Unit
Investigators Dr. Joan Albert Arnaiz jaarnaiz@clinic.ub.es
00 34 932279838
Judit Pich jpich@clinic.ub.es
00 34 932275400 (ext 2815)
Hospital Clinic Clinical Trials Unit
Villarroel, 170
08036 Barcelona, Spain Fax: 00 34 932279877

MVA B will be supplied by
Impfstoffwerk Dessau-Tornau GmbH (IDT)
Streetzer Weg 15a
Rodleben OT Torna
D-06862
Germany

The trial will be sponsored by

Fundació Clínic per a la Recerca Biomèdica
Dr. Josep M Gatell.Infectious Diseases Unit
Hospital Clínic
Villarroel, 170
08036 Barcelona, Spain
gatell0@attglobal.net
00 34932275430
Fax: 00 34 934514438

Investigations will be conducted in the following Immunology Laboratories

Cellular responses and flow cytometry:

Dr. Montserrat Plana
mplana@ub.edu
00 34 932275400 (ext 2884)
AIDS Research Laboratory
Hospital Clinic
Villarroel, 170
08036 Barcelona, Spain
Fax: 00 34 934514438

Antibody responses

Dr Mariano Esteban
mesteban@cnb.uam.es
00 34 915854553
Departamento de Biología Molecular y Celular
Centro Nacional de Biotecnología
Campus Universidad Autónoma. Cantoblanco.
28049-Madrid. Spain
Fax: 00 34 915866621

Version 2.0, 27/10/2011

EudraCT: 2009-016578-34

I. Introduction	4
II Summary of Trial	14
III Allocation of immunisations	16
IV Scientific questions to be addressed and analyses	16
1. General plan	17
1.1 Objectives	17
1.2 Design	17
1.3 Population	17
1.4 Trial products	18
1.5 End-points	20
2. Schedule of Visits	20
2.1 Recruitment	20
2.2 Screening visit	20
2.3 Vaccination visits	21
2.4 Follow up visits	21
2.5 Study schedule	22
3. Assessment of safety	23
4. Assessments of efficacy	24
5. Concomitant medication	24
6. Clinical study sites	24
7. Adverse events	25
7.1 Definitions	25
7.2 Relationship to study product	25
7.3 Reporting adverse events	25
7.4 Clinical management	26
8. Management of the trial	26
8.1 Data management at the Clinical Centres	26
8.2 Data management in the immunology laboratories	27
8.3 Data management at the Hospital Clinic CTU	27
8.4 Monitoring by HOSPITAL CLÍNIC CTU	28
8.5 Data Ownership	28
8.6 Trial Coordinating Committee (TCC)	28
8.7 Data and Safety Monitoring Committee (DSMC)	29
9. Statistical considerations	29
9.1 Sample size	29
9.2 Analysis	30
10. Confidentiality, ethics and responsibilities, including indemnity	30
11 Publication	31

Appendix I Case Report Form
Appendix II Patient Information Sheet and Informed Consent
Appendix III Investigator Agreement
Appendix IV Declaration of Helsinki
Appendix V SUSARs notification form
Appendix VI Grading of clinical and laboratory adverse events
Appendix VII MVA-B IMPD
Appendix VIII Financial Terms
Appendix IX Insurance document

I. Introduction

(i) General introduction

Although the introduction of Highly Active Antiretroviral Therapy (HAART) in HIV therapy has declined the morbidity and mortality dramatically(*1*), severe drawbacks limit the clinical management of HIV-infected patients, involving metabolic complications and increased risks for cardiovascular diseases (*2*), therapy failure and accumulation of drug resistance mutations over time in approximately 50% of patients treated with HAART. Strategies to limit time on therapy are however rapidly followed by virus relapses within about three weeks in chronically infected patients, even if they were successfully treated with HAART before treatment interruption(*3-6*). These relapses reflect the lack of restoration of a protective immunity against HIV due to the reduction of HIV antigen production during therapeutic control of HIV(*7*).

(ii) HIV-1 vaccines

HIV-1 vaccine design has shown to be very difficult for several reasons. An effective *preventive* HIV-1 vaccine should be effective against (preferably) all HIV-1 subtypes. Furthermore, the vaccine should target epitopes that are relative well conserved because of the high mutation rate of HIV-1. The vaccine would have to deal with mucosal and haematogenic routes of transmission, and elicit both an adequate neutralizing antibody response and a cellular immune response, in order to be effective against both cell-free HIV-1 particles and HIV-1 infected cells(*8*).
Several vaccine strategies are currently being developed. Plasmid DNA has shown only limited immunogenicity in humans ***(9)***. Replication-incompetent adenoviruses are being tested in humans without demonstrating effectiveness (*10*) (*11*), in fact, it is possible that previous immunity to vector could favor HIV infection in vaccine recipients. Clinical trials with envelope subunits have not demonstrated effectiveness, although have been able to elicit neutralizing antibody responses. Recently, it has been reported in mass media that a combination of two vaccines a prime with 4 doses of ALVAC (canarypox) and a boost with 2 doses of monomeric gp120 used as a preventive vaccine has reduced HIV infections in 31% of vaccine recipients. This combination was not able to reduce viral load or change CD4+ T cell count outcome. These data will be reported in next AIDS Vaccine Congress to be held in Paris next October 19th to 22nd 2009. These data open new questions. This is the first time that a HIV vaccine has demonstrated effectiveness. It should be investigated which surrogate markers are responsible of HIV prevention (neutralizing antibodies, cellular immunity, mucose immunity or a combination of these responses). In addition, it should be important to know why these responses were not able to change viral load set-point or CD4+ T cell count outcome, it suggest that immunity responsible of prevention could be different than those responsible of progression of the disease. Other live, recombinant vectors like modified vaccinia (pox) viruses like NYVAC and MVA seem to be promising. NYVAC has been tested both in human and animal models (*12-14*) with good results, a phase II clinical trial has been designed to be berformed in the next future (AFREVACC).
Because of these difficulties in HIV-1 vaccine design, it seems reasonable to investigate whether the current available vaccines could be used therapeutically, i.e. in patients already infected with HIV-1(*15*). The aim of therapeutic vaccination would be to enhance the HIV-1 specific immunity, allowing an interruption of antiretroviral treatment, limiting toxicity, resistance development and costs. Although several clinical phase-1 and 2 studies have been done with HIV-1 specific vaccines in HIV-1 infected subjects, only a few studies(*16-18*) have shown efficacy compared to a control group in the setting of an interruption of antiretroviral treatment (i.e. change in the setpoint of plasma HIV-1 RNA after treatment interruption and time off treatment). Two of these studies(*16, 17*) (one performed by our group) used a vaccine based on autologous dendritic cells pulsed by attenuated autologous HIV-1 isolates. Other 2 studies have demonstrated limited effectiveness. One (*18*) used a canarypox based vaccine combined with interleukin-2; the other recombinant MVA(*19*).

Although these results are encouraging, it must be stressed that these effects didn't occur in all vaccinees and that the effects were not lasting. Therefore, further research on determinants of efficacy, type and delivery of vaccines is necessary.

(iii) MVA-B

MVA

The Modified Vaccinia virus Ankara (MVA) has been developed towards the end of the smallpox eradication campaign in the seventies of the previous century, to obtain a vaccine with a better safety profile than the vaccines that were in use at that time. MVA was derived from Vaccinia strain Ankara, by over 570 passages in chicken embryo fibroblast cells (CEF) (*20*). The complete genomic sequence has been sequenced and has a length of 178 kb. It consists of 193 open reading frames (ORFs), corresponding to 177 genes, of which 25 are split and/or have suffered mutations resulting in truncated proteins. These numerous mutations, affecting host interactive proteins and some structural proteins, explain the attenuated phenotype of MVA(*21*).

The resulting MVA strain had lost the capacity to productively infect mammalian cells and suffered six major deletions of DNA, totaling 31,000 base pairs. MVA was proven to be avirulent even in immunosuppressed animals. There is clinical experience with MVA as vaccine against smallpox in over 120,000 people(*22, 23*). During extensive field studies, including high risk patients, no side effects were associated with the use of the MVA vaccine(*22, 23*). The combination of a very good safety profile and the ability to deliver antigens in a highly immunogenic way makes MVA suitable as vaccine vector. MVA can stimulate antibody and T cell responses even in presence to pre-existing antibodies(*24*). MVA has shown to be effective in primates and humans for several viruses, including SIV and SHIV(*25-29*).

MVA-B

Inserts

The HIV genes expressed in the recombinant vector are derived from the HIV-1 natural isolate BX08 (for gp120) and Gag-Pol-Nef from HIV-IIIB. All HIV genes have been optimised for codon usage since it has recently been shown that humanisation of synthetic HIV gene codons allowed for an enhanced and REV/RRE- independent expression of *env* and *gag-pol* genes in mammalian cells. Genes were optimised for both safety and translation efficiency.

The *env* gene has been designed to express the secreted gp120 form of the envelope proteins and contain an optimal synthetic leader sequence for enhanced expression. The *gag, pol* and *nef* genes were fused to express a Gag-Pol-Nef polyprotein. An artificial -1 frameshift introduced in the natural slippery sequence of the *p7-p6* gene junction results in an in-frame Gag-Pol-Nef fusion protein due to the absence of ribosomal frameshift. An N-terminal Gly $\rightarrow$ Ala substitution in *gag* prevents the formation and release of virus-like particles from transfected cells. This strategy should allow for an equimolar production of Gag, Pol and Nef proteins and an enhanced MHC Class-I restricted presentation of their CTL epitopes.

For safety and regulatory reason, the packaging signal sequence has been removed; protease active site mutated; the integrase gene deleted; and the reverse transcriptase gene disrupted by insertion of a scrambled *nef* gene at the 3' end of the DNA sequence coding for the RT active site known to be associated with an immunodominant CTL epitope. The *nef* gene has been dislocated by fusing its 5' half to its 3' half without losing its immunodominant CTL epitopes.

Plasmids pMA60gp120B/gagpolnefB-12,17 and pLZAW1 were provided by Sanofi-Pasteur. A 5.6 kbp DNA fragment containing the two synthetic early/late (E/L) promoters in a back-to-back orientation individually driving a codon optimized $_{BX08}$gp120 and $_{IIIB}$GPN genes of HIV-1 clade B was excised from plasmid pMA60gp120B/gagpolnefB-12,17 with the restriction endonuclease BamHI. The insert was modified by incubation with Klenow DNA polymerase to generate blunt ends, and inserted into the pLZAW1 vector (previously digested with AscI, modified by incubation with Klenow, and dephosphorylated by incubation with Alkaline Phosphatase, Calf Intestinal (CIP) generating the plasmid transfer vector pLZAW1gp120B/gagpolnef-B-1. The resulting plasmid directs the insertion of the foreign genes into the TK locus of MVA genome and allows the generation of a recombinant virus without selectable marker.

Construction of the recombinant virus MVA-B

Primary chick embryo fibroblast cells (CEF) from 11-day old SPF eggs were infected with MVA at a multiplicity of 0.05 PFU/cell and then transfected with 10 □g DNA of plasmid pLZAW1gp120B/gagpolnef-B-1 using lipofectamine reagent according to the manufacturer´s protocol (Invitrogen, San Diego, CA). After 72 h post infection the cells were harvested, sonicated and used for recombinant virus screening. Recombinant MVA viruses containing the $_{BX08}$gp120 and $_{IIIB}$Gag-Pol-Nef genes from clade B, and transiently co-expressing the □-gal marker gene (MVA-B (X-gal+)), were selected by consecutive rounds of plaque purification in CEF cells stained with 5-bromo-4-chloro-3-indolyl β-galactoside (X-Gal) (300 □g/mL). In the following plaque purification steps, recombinant MVA viruses containing the $_{BX08}$gp120 and $_{IIIB}$Gag-Pol-Nef genes and having deleted the □-gal gene (by homologous recombination between the TK left arm and the short TK left arm repeat that are flanking the marker) were isolated by two additional consecutive rounds of plaque purification screening for non-staining viral foci in CEF cells in the presence of X-Gal (300 µg/mL). In each round of purification the isolated plaques were expanded in CEF cells for 3 days, and the crude virus obtained were used for the next plaque purification round. The resulting MVA-B virus was grown in CEF, purified through two 45% (w/v) sucrose cushions and titrated by immunostaining. Purity of the recombinant virus was confirmed by PCR with primers spanning the junction and internal regions of the inserts and by DNA sequence analysis.

The multigenic recombinant MVA-B (env, gag, pol, nef clade C) has been shown to be stable for the HIV genes after 10 passages.

Non-clinical studies with HIV-1 specific recombinant MVA-B vaccines

Toxicity and biodistribution

The MVA-B vaccine used in this clinical study has been tested for toxicity and biodistribution in rats (unpublished study report by Convance Laboratories Ltd, Harrogate, UK). In this study 40 rats (20 male and 20 female) received three intramuscular administrations (three doses at 14 day intervals) of 0.4 mL vaccine (0.2 mL into each calf muscle), 1x10^{8} pfu/mL, which far exceeds the equivalent dose that will be used in this phase-1 clinical study. A control group was injected with the same volume of saline on the same time points. Of each group, ten animals were necropsied on Day 30 and ten animals were necropsied on day 51 (of each 10 5 were examined for toxicity and 5 for PCR on MVA-B).

There were no unscheduled deaths after administration of MVA-B. There were no treatment-related clinical signs. Body weight and food consumption were unaffected by treatment. No effect of treatment was detected by the ophthalmoscopic examinations. Increases in neutrophils of 2.3 and 4.3 fold were recorded for treated males and females respectively on day 30 (24 hours after completion of the third dosing). By day 51 (22 days after the final dose) the vaccinated animals had circulating levels of neutrophils similar to

controls. There was no effect of treatment on clinical chemistry parameters. Organ weights were unaffected by treatment.

At the day 30 kill, there were no macroscopic findings due to either local or systemic effects of the MVA B HIV vaccine. Microscopically, there was a minor increase in the severity of inflammatory cell foci/ myositis at the injection sites of treated animals compared with controls, suggestive of a minor local effect due to the MVA-B vaccine. There were no microscopic findings suggestive of systemic effects due to the MVA B HIV vaccine. At the day 51 kill, there was evidence of almost complete reversal of the injection site findings seen in treated animals at the day 30 kill. In other tissues in treated animals, there were no microscopic findings suggestive of delayed systemic effects due to the MVA B HIV vaccine.

Furthermore, other HIV-1 specific recombinant MVA vaccines have been evaluated for safety and biodistribution in animal studies. Biodistribution and safety of the HIV-1 specific recombinant MVA vaccine MVA-HIVA expressing HIV-1 clade A *gag* p24/p17 sequences (the same vaccine as used in the clinical trial cited below (Mwau et al. 911-19) was examined in rhesus macaques infected with SIV (SIVmac32H or SIVmac220) and in SCID (severe combined immunodeficiency) mice (Hanke et al. 1507-14). The macaques were vaccinated with a single dose of 5 x 10^7 pfu intradermally, and the mice with 5 x 10^6 pfu intradermally on days 0 and 15. In the macaques, after intradermal administration of MVA·HIVA no organ tropism was detected that could correlate with target organ toxicity, since no MVA-HIVA DNA could be detected in any of the examined organs (testes, epididymis, ovary, blood, brain, heart, spleen, kidney, liver, mesenteric lymph nodes, draining axillary lymph nodes, skin and muscle at injection site). In particular, no positive signal was found in any gonad sample from male or female animals, so the risk of transfer of vaccine DNA into the germ cells using this route of administration is regarded to be negligible.

Similarly to SIV-infected rhesus macaques, intradermal administration of MVA·HIVA into severely immunodeficient mice lacking T and B cell responses has not posed a significant safety concern nor it increased a risk for persistence of the vaccine-derived nucleic acid sequences. A positive PCR signal in the vaccinated SCID mice was observed only at the earliest examined timepoint, 49 days after the second vaccination, in 4 out of 6 skin samples at the injection site. This results are consistent with earlier results with the same vaccine in non-immunodeficient mice (Hanke et al. 108-14) and the safety results of MVA in severely immunocompromized cynomolgus macaques (Stittelaar et al. 3700-09).

Immunogenicity and challenge data in animal models

Mice inoculated with MVA-B in prime/boost protocols with the homologous vector or with heterologous vector, like DNA-B (two plasmid vectors that express either gp120 or Gag-Pol-Nef, same inserts as for MVA-B) induced specific immune responses against the four HIV antigens in both Balb/c and humanized HLA-A2 mice (*30*). Moreover, a similar MVA construct but expressing the HIV-1 89.6p (gp120) and the poliprotein Gag-Pol-Nef of SIVmac239 induced in macaques specific immune responses to the four HIV-SIV immunogenes and trigger high and long-term protection following challenge with pathogenic SHIV89.6p (*37*).

There is other efficacy data of several animal studies with other HIV-1 or SIV specific recombinant MVA vaccines. The key studies with SIV specific recombinant MVA vaccines in macaques are summarized in table 1. No safety problems were reported in these studies, although only the first study (Hirsch et al. 3741-52) explicitly mentioned the absence of side effects after vaccination with MVA. These studies reported CTL responses (Seth et al. 10112-16;Seth et al. 2502-09;Barouch et al. 5151-58), neutralizing antibody responses (Barouch et al. 5151-58;Ourmanov et al. 2960-65;Ourmanov et al. 2740-51), lower plasma SIV RNA levels(Hirsch et al. 3741-52;Seth et al. 2502-09;Ourmanov et al. 2960-65;Barouch et al. 5151-58) and even prolonged survival(Ourmanov et al. 2960-65) in vaccinated animals.

Other studies with HIV-1 specific recombinant MVA vaccines in animals have been done to examine to compare the use of MVA alone with a combination of an MVA vaccine with a protein (Earl et al. 270-81) or DNA vaccine (Amara et al. 7625-31;Amara et al. 1949-55). In these studies, there was no statistical significant difference between the efficacy of MVA alone and the combination with a protein or DNA vaccine. In a study comparing an MVA based vaccine with live attenuated SIVmac1A11 in infant rhesus macaques, no significant difference was found in the (modest) protection after oral challenge with SIVmac251(Van Rompay et al. 179-90).

Others studied the combination of priming with a DNA vaccine (Ellenberger et al. 21-32) (Amara et al. 124-33) (Makitalo et al. 2407-19;Wang et al. 846-59) (Wee et al. 75-80) (Smith et al. 140-44;Smith et al. 1335-47;Smith et al. 654-65) (Su et al. 637-48;Gherardi et al. 6209-20) (Bertley et al. 3745-57), or both a protein and a DNA vaccine (Buge et al. 891-900), or a viral vector other than MVA (Ramsburg et al. 3930-40), boosted with a recombinant MVA based vaccine. Furthermore, the following mucosal administration routes have been examined (not comparative): rectal (Makitalo et al. 2407-19;Wang et al. 846-59), intranasal (Gherardi et al. 6209-20;Bertley et al. 3745-57) and oral (Makitalo et al. 2407-19).

In none of these studies safety problems associated with MVA have been reported.

Table 1: Summary of key studies with SIV specific recombinant MVA vaccines in macaques

Study/ reference	Vaccine	Animals	Design	Safety	Efficacy
Hirsch et al. 1996(Hirsch et al. 3741-52)	Recombinant MVA expressing SIVsm-H4 *gag-pol, env* or non-recombinant MVA<br>Dose: 5 x 10^8 $TCID_{50}$, I.M.	12 rhesus monkeys (M. mulatta)<br>4 vaccinated with recombinant MVA<br>2 vaccinated with non-recombinant MVA<br>6 vaccinated with other recombinant (n=4) or non-recombinant (n=2) vaccinia vaccine (Wyeth-SIV)	4 vaccinations: week 0, 12, 20, 28.<br>Challenge: SIVsmE660 at week 32.	pox lesions in animals vaccinated with Wyeth-SIV, no side effects in animals vaccinated with MVA	low levels of viremia in 3 of 4 animals vaccinated with recombinant MVA, and in 1 of 4 animals vaccinated with Wyeth-SIV
Seth et al. 1998(Seth et al. 10112-16)	Recombinant MVA expressing SIVsm-H4 *gag-pol* or non-recombinant MVA<br>Dose: 10^8 pfu, I.M.	6 rhesus monkeys (M. mulatta; expressing major histocompatibility complex Mamu-A*01)<br>4 vaccinated with recombinant MVA<br>2 vaccinated with	2 vaccinations: at week 0 and 13.<br>No challenge.	No safety problems reported	*gag*-specific CTL response in animals vaccinated with recombinant MVA

		non-recombinant MVA			
Ourmanov et al. 2000(Ourmanov et al. 2960-65;Ourmanov et al. 2960-65)	Recombinant MVA expressing SIVsm-H4 *env* or *gag-pol* or *env-gag-pol* or non-recombinant MVA<br>Dose: 10^8 pfu, I.M.	24 rhesus monkeys (M. mulatta)<br>6 vaccinated with recombinant MVA-*env*<br>6 vaccinated with recombinant MVA-*gag-pol*<br>6 vaccinated with recombinant MVA-*env-gag-pol*<br>6 vaccinated with non-recombinant MVA	4 vaccinations: week 0, 4, 16, 28<br>Challenge: SIVsmE660 at week 32.	No safety problems reported	lower virus loads and prolonged survival in animals vaccinated with the recombinant MVA vaccines compared to control animals.<br>No statistical difference in survival and level of viral suppression between the three recombinant MVA vaccines;<br>low and non-lasting titers of neutralizing antibodies only in animals vaccinated with *env* containing vaccine
Seth et al. 2000(Seth et al. 2502-09)	Recombinant MVA expressing SIVsm-H4 *gag-pol* or non-recombinant MVA<br>Dose: 10^8 pfu, I.M.	6 rhesus monkeys (M. mulatta; expressing major histocompatibility complex Mamu-A*01)<br>4 vaccinated (4x) with recombinant MVA<br>4 vaccinated (1x)with recombinant MVA<br>2 vaccinated (4x) with non-recombinant MVA	4 vaccinations: week 0, 13, 35, 52 *or* 1 vaccination at week 52.<br>Challenge: SIVsmE660 at week 56.	No safety problems reported	*gag*-specific CTL response in animals vaccinated with recombinant MVA; lower plasma viral load setpoints in animals vaccinated with recombinant MVA vaccine compared to controls, correlated to CTL response
Barouch et al. 2001(Barouch et al. 5151-58)	Recombinant MVA expressing SIVmac239 *gag-pol* and HIV-1 89.6 *env* or non-recombinant MVA<br>Dose: 10^8 pfu, I.M.	8 rhesus monkeys (M. mulatta; expressing major histocompatibility complex Mamu-A*01)<br>4 vaccinated with recombinant MVA<br>4 vaccinated with non-recombinant MVA	3 vaccinations: week 0, 4, 21.<br>Challenge: SHIV-89.6P at week 27.	No safety problems reported	In 4 animals vaccinated with recombinant MVA: high-frequency secondary CTL response, high titer secondary and rapid emerging SHIV-89.6P-specific neutralizing antibodies, partial preservation of $CD4^+$ T cell count, reduced setpoint of

					of plasma viral load which was correlated to CTL-response.

Clinical studies with HIV-1 specific recombinant MVA vaccines

This clinical trial will be the third experience in humans with MVA-B. There are data of safety (not of immunogenicity due to samples problems) in Theravacc study in 10 chronic HIV infected patients on antirretroviral therapy. Patients included in Theravacc study received MVA-B in week 0 and 4 and no severe adverse effects were reported, 4 patients had mild side effects consisting in local pain at injected site or fever 24h following the vaccination. There were no changes in general analysis, CD4+ T cell count or virologic failures. Therefore, it could be concluded that MVA-B was safe in HIV infected patients. Data have not been published, but have been included in the IMPD when RISVAC02 was approved. RISVAC02 is the second experience in humans. This is a phase I clinical trial using MVA-B as a preventive vaccine and has a very similar design to the present study. In RISVAC02 has been vaccinated all the volunteers with 3 doses at the same Schedule proposed in this trial without any severe side effects. Clinical data will be presented at the end of 2010.

There is experience with other HIV-1 specific recombinant MVA vaccines in humans. A summary of these studies can be found in Table 2. In brief, two studies (Mwau et al. 911-19) (Goonetilleke et al. 4717-28) enrolled together 33 volunteers who received 1 or 2 vaccinations with an HIV-1 specific recombinant MVA vaccine.

Safety

DNA- and modified virus Ankara (MVA)-vectored candidate vaccines expressing human immunodeficiency virus type 1 (HIV-1) clade A-derived p24/p17 gag fused to a string of HLA class I epitopes, called HIVA, were tested in phase I trials in healthy, HIV-1/2-uninfected adults in Oxford, United Kingdom. Eight volunteers received MVA.HIVA (IAVI-003) alone and 9 volunteers from study IAVI-001 were boosted with MVA.HIVA 9–14 months after DNA priming (IAVI-005). Immunogenicity results observed in these trials were published previously (Mwau et al. 911-19). Overall, both candidate vaccines were safe and well tolerated. There were no reported vaccine-related adverse events over the 6-month period of the study and up to 2 years after the last vaccination. There were no moderate or severe local symptoms recorded after the pTHr.HIVA DNA intramuscular administration. Almost all participants experienced local reactogenicity events such as redness and induration after MVA.HIVA intradermal injection (Cebere et al. 417-25).

The researchers of the study with healthy volunteers(Mwau et al. 911-19) mention that in a (still unpublished) next study in which more than 100 healthy volunteers have been vaccinated no such reactions have been seen similar safety results were seen after 200 immunizations with a recombinant MVA vaccine in 100 volunteers.

Efficacy

Induction of CD4+ and CD8+ T cell responses have been reported in both studies (Mwau et al. 911-19) (Goonetilleke et al. 4717-28).
The immunogenicities of candidate DNA- and modified vaccinia virus Ankara (MVA)-vectored human immunodeficiency virus (HIV) vaccines were evaluated on their own and in a prime–boost regimen in phase I clinical trials in healthy uninfected individuals in the United Kingdom (Mwau et al. 911-19). The vaccines expressed a common immunogen, HIVA, which consists of consensus HIV-1 clade A Gag p24/p17 proteins fused to a string of clade A-derived epitopes recognized by cytotoxic T lymphocytes (CTLs). Both the DNA and the MVA vaccines alone and in a DNA prime–MVA boost combination were safe and induced HIV-specific responses in 14 out of 18, seven out of eight and eight out of nine volunteers, respectively.

A double-blind randomized phase I trial was conducted in human immunodeficiency virus type 1 (HIV-1)-negative subjects receiving vaccines vectored by plasmid DNA and modified vaccinia virus Ankara (MVA) expressing HIV-1 p24/p17 gag linked to a string of CD8_ T-cell epitopes. The trial had two groups. One group received either two doses of MVA.HIVA (2x MVA.HIVA) (*n* = 8) or two doses of placebo (2x placebo) (*n* =4). The second group received 2x pTHr.HIVA followed by one dose of MVA.HIVA (*n* = 8) or 3x placebo (*n*=4). In the pTHr.HIVA-MVA.HIVA group, HIV-1-specific T-cell responses peaked 1 week after MVA.HIVA vaccination in both ex vivo gamma interferon (IFN-gamma) ELISPOT (group mean, 210 spot-forming cells/106 cells) and proliferation (group mean stimulation index, 37), with assays detecting positive responses in four out of eight and five out of eight subjects, respectively. No HIV-1-specific T-cell responses were detected in either assay in the 2x MVA.HIVA group or subjects receiving placebo. Using a highly sensitive and reproducible cultured IFN-gamma ELISPOT assay, positive responses mainly mediated by CD4 T cells were detected in eight out of eight vaccinees in the pTHr.HIVA-MVA.HIVA group and four out of eight vaccinees in the 2x MVA.HIVA group. Importantly, no false-positive responses were detected in the eight subjects receiving placebo. Of the 12 responders, 11 developed responses to previously identified immunodominant CD4 T-cell epitopes, with 6 volunteers having responses to more than one epitope. Five out of 12 responders also developed CD8 T-cell responses to the epitope string. Induced T cells produced a variety of anti-viral cytokines, including tumor necrosis factor alpha and macrophage inflammatory protein 1beta.

Table 2: Summary of clinical studies with HIV-1 specific recombinant MVA vaccines

Study/ reference	Vaccine	Subjects	Design	Safety	Efficacy
Mwau et al. 2004(Mwau et al. 911-19) (Cebere et al. 417-25)	recombinant MVA expressing “HIVA”: HIV-1 clade A *gag* p24/p17 sequences, and a DNA vaccine expressing the same proteins<br><br>dose: 5 x 10^7 pfu MVA-HIVA , intradermally (and DNA-HIV 100 or 500 µg I.M.)	17 healthy volunteers uninfected with HIV-1 (and low risk of infection)	8 volunteers received 2 vaccinations : at week 0 and 3 (MVA-HIVA only) and 9 volunteers received DNA vaccine at week 0 and 3, followed by MVA boost on 9 to 14 months after their last dose of DNA-HIVA.<br><br>(No challenge).	Local reactions; erythema, induration, very small blister like lesions (1-2 mm) that never coalesced or broke.<br><br>Systemic reactions: “minimal”, except 1 subject who developed a febrile illness with vomiting one day after the first vaccination, relation with vaccine unclear (similar safety results reported by the authors (unpublished) after 200 immunizations with MVA-HIVA in 100 volunteers)	HIV-specific T cell responses in 7/8 subjects immunized with MVA-HIVA alone (detectable after 1 year in 5 subjects) and in 8/9 subjects who had received the DNA-prime MVA-boost immunizations (responses in subjects vaccinated with MVA were stronger than in subjects immunized with DNA alone).
Goonetilleke et al 2006 (Goonetilleke et al. 4717-28)	pTHr.HIVA and MVA.HIVA Both vectors express the HIV-1 clade A consensus sequence gag p24 and p17 coupled to a string of partially overlapping CD8 T-cell epitopes, which are derived from the gag, pol, nef, and env proteins and are restricted by 17 different HLA class I alleles.	16 healthy volunteers uninfected with HIV-1 (and low risk of infection)	The trial had two groups. One group received either two doses of MVA.HIVA (2xMVA.HIVA) (*n* = 8) or two doses of placebo (2xplacebo) (*n* =4). The second group received 2_ pTHr.HIVA followed by one dose of MVA.HIVA (*n* = 8) or 3x placebo (*n*= 4).	Not described	Using a highly sensitive and reproducible cultured IFN-gamma ELISPOT assay, positive responses mainly mediated by CD4 T cells were detected in eight out of eight vaccinees in the pTHr.HIVA-MVA.HIVA group and four out of eight vaccinees in the 2x MVA.HIVA group.

	dose: 10^8 pfu MVA-HIVA , intradermal				

(iv). Rationale for this study

This proposal to study a modified pox viral vector is innovative in the following:

a. HIV subtype B accounts for the most frequent virus strain in Europe and North America, as well as in many parts of the world.
b. This novel vaccinia construct expressing HIV subtype B gag, pol, env and nef antigens is to be studied in humans for the 3rd time and is the first time to have immunological data in HIV infected patients.

II Summary of Trial

30 treated chronic HIV-1 infected patients with $CD4^+$ cell counts above 450 cells/ mm^3 will be randomized 1:2 to receive placebo (n=10) or vaccine (n=20) at week 0, 4 and 16 and will be observed at the Investigation Unit of the study site for one hour following vaccination.

MVA HIV-B n=20
placebo n=10

Participants, clinical investigators and cellular immunologists will be blind to the allocation.

Study visits will be (see flow chart) at week -4 (screening), weeks 0 (vaccination 1), 1, 2, 4 (vaccination 2) and at week 5, 6, 8, 16 (vaccination 3), 17, 18, 20 and 24, when HAART will be interrupted. After interruption, controls will be done at week 26, 28, 32, 36 and 48,

A diary card will be given (see Appendix), with instructions and a full verbal explanation, for participants to record local and systemic adverse events following one week after immunisation.

The three centres are:
- Hospital Clinic, Barcelona
- Hospital Germans Trias i Pujol, Badalona and
- Hospital Gregorio Marañón, Madrid

Primary objective: to evaluate the safety of MVA-B administered by an IM injection on week 0, 4 and 16 in HIV-1 infected patients during concomitant successful antiretroviral therapy, during the first 24 weeks (4 weeks after the second vaccination). The evaluation of the HIV-1 specific immunity after 1, 2 and 3 vaccinations with MVA-B in HIV-infected patients successfully treated with HAART;

Secondary objectives: to evaluate the viral load control after an analytical interruption of HAART.
The data management and analysis will be coordinated by the Hospital Clinic Clinical Trials Unit and the randomisation and serious adverse event reporting number is:

00 34 932275400 (ext 2815)

The trial will be overseen by a Trial Coordinating Committee which will include the Principal Investigators from each centre, and two independent members including the Chair. A Data and Safety Monitoring Committee will also be appointed to review the design and protocol, but will only meet during the trial if 3 or more participants experience an unexplained, unexpected grade 3 or 4 clinical or laboratory event (confirmed on attendance or repeat testing), not resolved within 72 hours and considered probably or possibly related to vaccine.

Internationally accepted good manufacturing and good clinical practices will be followed. Screening will not commence until the ethical approval local to the centre is obtained and immunisations will not commence until the regulatory approval appropriate to the clinical centre is in place.

III Allocation of immunisations

The products will be in liquid form and must be stored at –20°C, and thawed at room temperature prior to use. The volume is 1ml in 2ml vials. The vials should be gently swirled but not inverted. The immunisations will be given into the non-dominant deltoid muscle.

Time in weeks			0	4	16
		**Number**			
**Vaccine groups**	1	20	~1x10^8 pfu/ml MVA-B*	~1x10^8 pfu/ml MVA-B	~1x10^8 pfu/ml MVA-B
**Control group***	2	10	placebo	placebo	placebo

* this is not a control group in the statistical sense; this group will act to reduce bias in the assessment of adverse events and ELISPOT assays.

IV Scientific questions to be addressed and analyses

Question	Groups	Primary safety end-points
Is the novel regimen safe?	1	Grade 3 or above adverse event within 28 days of any vaccination expressed as a proportion with confidence intervals
		**Primary immunogenicity end-points:**
How immunogenic is the MVA-B against clade B peptides?	1	ELISPOT responses following immunisations to pools of B peptides during this trial

Detailed study schedule in section 2.5

1. General plan

1.1 Objectives

Primary objective: to evaluate the safety of MVA-B administered by an IM injection on week 0, 4 and 16 in HIV-1 infected patients during concomitant successful antiretroviral therapy, during the first 24 weeks (4 weeks after the second vaccination). The evaluation of the HIV-1 specific immunity after 1, 2 and 3 vaccinations with MVA-B in HIV-infected patients successfully treated with HAART;

Secondary objectives: to evaluate the viral load control after an analytical interruption of HAART.

1.2 Design

The design includes randomisation to a matched placebo control, but the purpose of the control group is to minimise observer bias in clinical and laboratory assessments, not to act as a parallel comparison in the analysis.

1.3 Population

The study will require 30 healthy male and female volunteers who are HIV infected through three clinical centres: Hospital Gregorio Marañón de Madrid, Spain; Hospital Germans Trias I Pujol, Badalona, Spain and Hospital Clinic, Barcelona, Spain.

Inclusion Criteria

1. Patient is ≥ 18 years of age;
2. Voluntarily signed informed consent;
3. Patient is male, or female with negative pregnancy test prior to enrolment;
4. Patient has a proven HIV-1 infection (with positive antibodies against HIV-1 and a detectable plasma HIV-1 RNA);
5. Patient must be on stable treatment with HAART for at least 6 months (HAART is defined as an antiretroviral regimen consisting of at least three registered antiretroviral agents*);
6. Mean of all measured $CD4^+$ cell counts during the 6 months prior to the start of HAART must be above or equal to 200 cells/ mm^3
7. Current $CD4^+$ cell count must be at least 450 cells/ mm^3;
8. HIV-RNA must be below 50 copies/ mL for the last 6 months prior to inclusion, during at least two measurements (occasional so called ‘blips’ up to 50 copies/mL are permitted);
9. Patient is one of the following:
   - not sexually active, **OR**
   - a heterosexually active female, agreeing to use condoms with her partner from 14 days prior to the first vaccination until 4 months after the last, even though using another method of contraception, and willing to undergo pregnancy tests during screening and prior to each vaccination, **OR**
   - a male, agreeing to use condoms with his partner from the day of the first vaccination until 4 months after the last vaccination.

* Low dose ritonavir used for boosting other protease inhibitors does not count as one of these three antiretroviral agents.

Exclusion Criteria

1. Treatment with a non-HAART regimen of antiretroviral agents prior to the start of HAART;
2. History of a CDC class C event (see Appendix);
3. Interruption of HAART during the course of the study which is expected at the time of inclusion;
4. History of exposure <20 years ago to any poxvirus based vaccine;
5. Patient is female and has a positive pregnancy test or the wish of pregnancy:
6. Active opportunistic infection, or any active infection or malignancy within 30 days prior to screening visit;
7. Therapy with immunomodulatory agents, including cytokines (e.g. IL2) and gamma globulin, or cytostatic chemotherapy within 90 days prior to screening visit;
8. History of allergy to any vaccine component;
9. Use of anti-coagulant medication;
10. Use of any investigational drug during the 90 days prior to study entry;
11. Previous failure to antiretroviral and/or mutations conferring genotypic resistance to antiretroviral therapy
12. Any other condition which, in the opinion of the investigator, may interfere with the evaluation of the study objectives.

Withdrawal and replacement criteria

A patient will prematurely discontinue from the study in case of withdrawal of Informed Consent or if the investigator considers it in the best interest of the patient to withdraw.

A patient will not receive the second or third vaccination if:
- the patient becomes pregnant during the study;
- there is a need to start concomitant treatment with medication that is not compatible with the use of the study vaccine;
- a severe adverse event occurs which is considered as a (possible) result of the vaccinations;
- it is in her or his best interest in the opinion of the investigator (e.g. interruption of HAART).

Patients who do not receive the second vaccination (for any reason) will be asked to stay in follow up for safety and immunological assessments according to the protocol where possible. In case of an adverse event subjects will be followed until the adverse event has been resolved or stabilized (until six months after last study visit at week 48). Safety data of these patients will be collected according to the protocol where possible.
Only patients who haven't received any of the study vaccinations will be replaced.
After interruption of antiretroviral therapy, this treatment will be reintroduced if CD4 T cell count drop below 350 cells/mm^3 or viral load increase above 75.000 copies/ml in two determinations at least 1 month apart. In addition, antiretroviral therapy will be reintroduced if the patients wish to reintroduce it, or there are any symptoms due to discontinuation (disease progression or acute retroviral syndrome).

1.4 Trial products

1.4.1 Supply, storage and composition of MVA-B and placebo

Impfstoffwerk Dessau-Tornau GmbH (IDT) is responsible for manufacture and supply of all clinical material of the vaccine according to Good Manufacturing Practice. The presentation is in a liquid form, 1ml in single dose 2ml vials, which should be stored at -20°C. The composition is below:

Ingredients	MVA HIV-B	Placebo
1. Active substances		
Modified Pox virus, strain MVA clade -B (expressing HIV-1 Bx08gp120 and IIIB gagpolnef)	~ 1 x 10^8 pfu/ml	--
2. Excipients		
Tris (hydroxymethyl)-amino methane	0.121 mg/ml	0.121 mg/ml
Sodium Chloride	0.818 mg/ml	0.818 mg/ml
3. Impurities		
Chicken Embryo Fibroblast protein	Traces	--
Gentamycin	≤ 20µg/ml	--

1.4.2 Preparations prior to use

Prior to use the vials should be thawed at room temperature. When completely thawed the vials should be gently swirled. Care must be taken not to invert the vials.

1.4.3 Labels

MVA-B vials will be labelled by IDT. The label will contain the information including but not limited to the clinical sites and principal investigators for each centre, the storage details and the name of the supplier for the product.

1.4.4 Dispensing records and disposal of unused product

The designated pharmacist will, upon receipt of supplies prior to commencement of the trial, conduct an inventory and complete a receipt, one copy of which will be retained at the site, one copy forwarded to Hospital Clinic CTU and the original returned to the supplier. During the trial the pharmacist will be responsible for reviewing the dispensing log.

On the day of immunisation, and with the participant present, the Investigator will complete a prescription with the trial number, date of birth, and immunisation number (1/2). The immunisation number and date will be entered against the trial number in the dispensing log. The vial label will be cross-checked against the details on the prescription and dispensing log by two individuals, prior to product being administered.

The individual who administers the injection will be responsible for ensuring that the return of the used vials is recorded in the dispensing log at the end of the clinical session, and that they are placed in the participant's carton.

At the end of the trial all used and unused vials will be checked against the inventory by staff from Hospital Clinic CTU before returning to the supplier or disposal on site according to local pharmacy guidelines and applicable regulations. Documentation of disposal will be provided to Hospital Clinic CTU and the supplier.

During the trial, product accountability will be monitored by the prescriptions, the dispensing log, the returns, the trial register and data collected on the case report forms.

1.5 End-points

1.5.1 Primary

The primary endpoints are safety and immunogenicity as defined below.

The primary safety parameters will be graded according to appendix 4, and are:

- Grade 3 or above local adverse event (pain, cutaneous reactions including induration)
- Grade 3 or above systemic adverse event (temperature, chills, headache, nausea, vomiting, malaise, and myalgia)
- Grade 3 or above other clinical or laboratory adverse event confirmed at examination or on repeat testing respectively
- Any event attributable to vaccine leading to discontinuation of the immunisation regimen

Data on local and systemic events listed above will be solicited with specific questions for a minimum of 7 days following each immunisation. Data on other clinical and laboratory events will be collected with an open question at each visit and through routine scheduled investigations respectively.

The primary immunogenicity parameters will be quantitative or present/absent, and are:

- cellular responses - CD8/CD4+ T cell responses (ELISPOT) at week 6/8, 18/20 and at any point following both immunisations

1.5.2 Secondary

Secondary safety and immunogenicity end-point information will be collected on all participants on the following:

- all grade 1 and 2 adverse events within 28 days of a vaccination
- viral load rebound after HAART interruption compared between both arms and with baseline viral load before any medication in each arm
- antibody responses
  - ∧ binding titration to the construct MVAB
  - ∧ binding titration to and neutralisation of vaccinia
- cellular responses
  - ∧ intracellular cytokine analysis at week 6, 18

2. Schedule of Visits

2.1 Recruitment

HIV infected patients will be recruited in day care centers of the 3 centers.

An overview of all study procedures can be found in the study Flow Chart.

2.2 Screening visit

A screening assessment for eligibility will be performed between week -4 and week -1.
Written informed consent must be obtained prior to the initiation of any study related intervention. Screening procedures include:

- demographic information: date of birth, sex, ethnic group;
- HIV-associated conditions (mode of infection, date of last negative and first positive HIV-1 test, nadir $CD4^+$ T cell count, history of antiretroviral treatment, current CDC status);
- relevant medical history; current medical conditions;
- history of smallpox (yes/ no and if applicable, age at which smallpox occurred) or vaccinia/ poxvirus based vaccination (yes/ no and if applicable, specify type of vaccination and age at which vaccination occurred);

- relevant medication history (all relevant medication up to 3 months prior to inclusion); co-medication reporting;
- height;
- body weight and vital signs: blood pressure, heart rate, body temperature;
- complete physical examination: general appearance, eyes, ears/ nose/ throat, cardiovascular, respiratory, gastrointestinal, neurological, musculoskeletal, skin, signs of smallpox vaccination, other;
- laboratory assessments: haematology and clinical chemistry, lymphocyte subsets (absolute and percentage $CD4^+$ and $CD8^+$ T cell counts), plasma HIV-1 RNA, hepatitis serology* (if not already known) and beta-HCG (in urine; female patients only);
- HIV-1 subtype determination: if subtype is unknown, it should be determined using the latest pre-study sample with detectable plasma HIV-1 RNA.

2.3 Vaccination visits

Patients will be vaccinated on weeks 0, 4 and 16 with one single dose of 10^8 pfu of the study vaccine. Prior to the injection for vaccinations 1 and 2 a physical examination will be done and blood samples (safety lab, immunology and virology, urine beta-HCG (female patients only), see below) will be taken. The vaccination will be administered by IM injection on weeks 0, 4 and 16 in respectively the upper right and the upper left arm, or vice versa. The injection site (right or left upper arm) will be registered for both vaccinations.
A plaster will be placed over the injection site 10 minutes after vaccination, for at least one hour. The plaster has to be transparent, so that the injection site can be observed, and impermeable and well attached, so that the spread of the vaccine virus will be prevented.

After vaccination on day 0 and day 28 patients will remain in the hospital for observation during the first hour following vaccination. Blood pressure, pulse and temperature will be measured before vaccination and at ½ and 1 hour after vaccination. After each vaccination all vaccinated patients will receive a diary to collect Vaccination related signs and symptoms during the first 7 days after vaccination (see below).

2.4 Follow up visits

Study visits will be (see flow chart) at week -4 (screening), weeks 0 (vaccination 1), 1, 2, 4 (vaccination 2) and at week 5, 6, 8, 16 (vaccination 3), 17, 18, 20 and 24, when HAART will be interrupted. After interruption, controls will be done at week 26, 28, 32, 36 and 48,
The interruption of efavirenz containing treatments will be as follows: 15 days before interruption, efavirenz will be changed to a protease inhibitor to avoid the risk of developing resistance due to different half-life of the drugs.

* HBs antigen, HBs antibodies, HBe antigen, HBe antibodies, HBc antibodies, HCV antibodies (in case of positive HCV antibodies: HCV PCR)

2.5 Study schedule

((X) indicates that a specimen will only be collected if indicated by history)

Visit Number	1	2	3	4	5	6	7	8	9	10	11	12	13	14	15	16	17	18
Nominal week (+/-week)	Up to -4	0	1	2	4	5	6	8	16	17	18	20	24	26	28	32	36	48
Immunisation		X			X				X									
STOP HAART													X					
Eligibility	X																	
History & exam[a]	X	X			X				X				X				X	X
Adverse event assessment		X	X	X	X	X	X	X	X	X	X	X	X	X	X	X	X	X
Haematology	X		X	X	X	X	X	X	X	X	X	X	X	X	X	X	X	X
Chem pathology	X		X	X	X	X	X	X	X	X	X	X	X	X	X	X	X	X
CD4 number & percentage	X		X	X	X	X	X	X	X	X	X	X	X	X	X	X	X	X
VIRAL LOAD	X		X	X	X	X	X	X	X	X	X	X	X	X	X	X	X	X
Cell storage		X		X			X				X		X	X			X	X
Store for HLA if needed		X																
Pregnancy test, if female		X			X				X									
Efficay assessment																		
Antibody responses	X						X				X		X	X			X	X
ELISPOT responses/ICC	X						X				X		X	X			X	X
Vaccinia response	X										X		X	X			X	X
Diary card		←	→		←	→			←	→								
Contact by trial staff		←	→		←	→			←	→								
TOTAL	110	50	16	46	16	16	116	16	16	16	156	16	146	116	16	16	116	116

[a]including weight in kg, height in cm and arm circumference in cm

3. Assessment of safety

- **Body weight and vital signs:** blood pressure, heart rate, body temperature (all visits).

- **Physical examination:** general appearance, eyes, ears/ nose/ throat, cardiovascular, respiratory, gastrointestinal, neurological, musculoskeletal, skin, other (screening, baseline, week 4 and 16, week 24, week 36, week 48, during all other visits only in case of adverse events).

- **Clinical symptoms**: current medical conditions and HIV-associated conditions (all visits), (S)AEs and SUSARs, (all visits except screening visit); (co)medication reporting (all visits).

- **Vaccination related signs and symptoms:**
  - The presence of the following signs and symptoms will be checked during all visits after vaccination 1 until week 24;
  - After the vaccination 1, 2 and 3 all vaccinated patients will receive a diary to collect the following vaccination related signs and symptoms 12 hours after vaccination and daily (preferably in the morning) during the first 7 days after vaccination.

  Local injection site reactions:
    - Pain at injection site;
    - Itching at injection site;
    - Redness/discoloration;
    - Fluid filled blisters;
    - Blood filled blisters;
    - Hard swelling in skin surface at or close to site.

  General/ systemic reactions:
    - Temperature (oral)*;
    - Chills/ rigors;
    - Malaise/ tiredness;
    - General muscle aches;
    - Headache;
    - Nausea;
    - Vomiting.

  All vaccination related signs and symptoms will be graded according to the table in Appendix.

- **Safety laboratory** (all visits):
  - Haematology: haemoglobin, MCV, leukocytes + differential, thrombocytes. Required amount of blood: 4 ml EDTA blood;
  - Chemistry: creatinine, ASAT, ALAT, alkaline phosphatase, γGT, total bilirubin, amylase, CPK, glucose, cholesterol, triglycerides. Required amount of blood: 4 ml heparinized blood.

- **Beta-HCG** (female patients only)
  - in urine (screening, weeks 0, 4 and 16).

- **Immunology**:
  - Lymphocyte subsets ($CD4^+$ and $CD8^+$ T cells, all visits). Required amount of blood: 4 mL EDTA blood (if blood for haematology is drawn, no extra blood has to be taken for $CD4^+$ and $CD8^+$ T cell count);
  - HLA typing (baseline). Required amount of blood: 10 ml of EDTA blood.

- PBMC storage Study visits will be (see flow chart) at week -4 (screening), weeks 0 (vaccination 1), 1, 2, 4 (vaccination 2) and at week 5, 6, 8, 16 (vaccination 3), 17, 18, 20 and 24, when HAART will be interrupted. After interruption, controls will be done at week 26, 28, 32, 36 and 48. Required amount of blood can be found in the laboratory manual.

- **Virology**:
  - Plasma HIV-1 RNA (all visits). Required amount of blood: 4 ml EDTA blood;

In case of an adverse event subjects will be followed until the adverse event has been resolved or stabilized (until 6 months after last study visit). Safety data of patients who have withdrawn Informed Consent will be collected according to the protocol where possible.

4. Assessments of efficacy

- **Immunology**:
  - HIV-1 specific $CD4^+$ and $CD8^+$ cells (at week -4 (screening), weeks 0 (vaccination 1), 1, 2, 4 (vaccination 2) and at week 5, 6, 8, 16 (vaccination 3), 17, 18, 20 and 24, when HAART will be interrupted. After interruption, controls will be done at week 26, 28, 32, 36 and 48). Required amount of heparinized blood (PBMC isolation for assessment of cellular response) and blood (serum storage for future assessment of HIV-1 specific humoral immune response) can be found in the laboratory manual;
  - Vaccinia-specific $CD4^+$ and $CD8^+$ cells and vaccinia-specific humoral immune response (screening, weeks 0, 4, 16 and week 20). Required amount of heparinized blood (PBMC isolation for assessment of cellular response) and blood (serum for assessment of humoral response) can be found in the laboratory manual.

5. Concomitant medication

Participating patients must have the intention to continue the HAART regimen at baseline during the course of the study program. If, for any reason, the HAART regimen is being changed or interrupted, the patient will remain in the study program, while documenting the changes and/ or interruption.

Co-medications, including drugs used for the primary or secondary prophylaxis of opportunistic infections, may be continued for the duration of the study (excluding immunomodulatory medication: immunosuppressive agents, or cytokines, or gamma globulin, or cytostatic chemotherapy). All medications have to be documented.

6. Clinical study sites

The three centres are:
- Hospital Clinic, Barcelona
- Hospital Germans Trias i Pujol, Badalona and
- Hospital Gregorio Marañón, Madrid

These centres have a large patient population of chronically HIV-1 infected patients eligible for this study program.

7. Adverse events

7.1 Definitions

An adverse event is any adverse experience occurring during the course of the study including the screening period.

Criteria for grading clinical and laboratory events are listed in appendix.

A severe adverse event is one **graded 3 or 4** by criteria in appendix 4. Some, but not all grade 3 and 4 adverse events will be "serious" by ICH GCP criteria below.

7.1.1 Serious Adverse Events (SAEs)

An adverse event is considered to be a "serious adverse event" by ICH Good Clinical Practice (ICH GCP) criteria if it results in the following:

- death,
- a threat to life,
- requires in-patient hospitalisation or prolongs existing hospitalisation (hospitalisation for elective treatment of a pre-existing condition is not included),
- results in persistent or significant disability or incapacity,
- is a congenital anomaly (i.e., the outcome of pregnancy involving a participant), or
- is any other important medical condition*.

*Examples of conditions regarded as "any other important medical condition" include allergic bronchospasm requiring intensive emergency treatment, seizures or blood dyscrasias which did not result in hospitalisation or development of drug dependency.

7.2 Relationship to study product

This can be classified as:

Unrelated adverse events that can be clearly explained by extraneous causes and for which there is no plausible association with study product, or adverse events for which there is no temporal relationship

Unlikely to be adverse events that may be temporally linked, but which are much more likely to be due to other causes than study product and which do not get worse with continuing use of product

Possibly adverse events that could equally well be explained by study product or other causes, which are usually temporally linked and may improve when not using study product but do not reappear when using study product

Probably adverse events that are temporally linked and for which the study product is more likely to be the explanation than other causes, which may improve when not using study product

Definitely adverse events that are temporally linked and for which the study product is the most likely explanation, which disappear or decrease when not using study product and reappear when using study product

7.3 Reporting adverse events

Adverse events should be recorded on the appropriate case record form and reported to the Hospital Clinic Clinical Trials Unit (CTU).

Any grade 3 or 4 adverse event (see appendix 4) or any event resulting in discontinuation of the vaccination schedule should be reported within 2 working days to the Hospital Clinic CTU of the decision to discontinue.

SAEs that are considered possibly, probably or definitely related should be reported to the Hospital Clinic CTU the same working day that the Clinical Investigator becomes aware of the event fulfilling the above criteria. This can be done by telephone or fax. The minimum criteria required in reporting a SAE are the participant identifiers (trial number/ date of birth/initials), reporting source (name of Investigator), and why the adverse event is identifiable as serious, and relationship to study product.

The adverse event reporting telephone number is 00 34 932275400 (ext 2815) and fax is 00 34 932279877

Staff at the Hospital Clinic CTU will confirm that the event qualifies as a Suspected Unexpected Adverse Drug Reaction (SUSAR) and arrange for urgent review of the case to take place within 2 working days. Those involved in this review will include the site Principal Investigator, the medical expert at CTU, and a clinician who is an independent member of the Trial Coordinating or Data Safety Monitoring Committee. The report will be prepared by the CTU Medical Expert and filed with the appropriate regulatory authorities, within the timelines required by national legislation. The CTU Medical Expert will inform the full Trial Coordinating and Data and Safety Monitoring Committees. The site Principal Investigator is responsible for notifying their Local Research Ethics Committee.

7.4 Clinical management

Events will be managed by the clinical trial team who will assess and treat the event as appropriate, including referral to an independent physician and/or the participant's General Practitioner if required. There will be clinical operating procedures in place for the management of abnormalities detected following urinalysis or routine laboratory tests.

7.4.1 Recommendations for discontinuation

Discontinuation of the study vaccine schedule due to an adverse event is at the discretion of the Clinical Investigator, but would be recommended in the event of a grade 3 or 4 clinical or laboratory event (confirmed on examination or repeat testing respectively) which did not resolve within 72 hours. There must be no further immunisations following:

1) extensive, indurated redness and swelling involving the major circumference of the arm, not resolving within 72 hours or
2) fever >= 39.5^{o}C within 48 hours; anaphylaxis; bronchospasm; laryngeal oedema; collapse; convulsions or encephalopathy within 72 hours

7.4.2 Unblinding

In the unlikely circumstances that a clinical or principal investigator needs to know which product a participant was allocated to in order to manage an adverse event, they must phone the Hospital Clinic CTU.

8. Management of the trial

8.1 Data management at the Clinical Centres

Hospital Clinic, Hospital Germans Trias I Pujol and Hospital Gregorio Marañón will be responsible for:

- Entering relevant information (see below) in the clinical notes, and holding a record for each participant which includes the bottom copy of the CRF with any changes made signed and dated

- The accurate completion of the case record forms
- The prompt return of the completed forms to the Hospital Clinic CTU.

Data will be recorded directly onto the case report forms, which will provide the majority of source data for the trial. There will be some additional source data in the clinical notes, such as medical history related to eligibility, dates visits including immunisation and details of clinical management (adverse events and concomitant medication).

Duplicate case record forms (CRFs) will be supplied by the Hospital Clinic CTU and the top copy returned to CTU for data entry after completion. Copies of laboratory reports containing the results of routine haematology, chemical pathology and immunology may be sent instead of completing these sections of the CRF, provided they are clearly labelled with the trial number and the date of collection. A member of the clinical trial team must sign the laboratory report. In the event of an abnormality, an indication should be given whether or not action was taken, the date of review and the signature of the clinician reviewing the result.

Changes to the CRF should be signed and dated, including changes made before the form is returned to the Hospital Clinic CTU.

CRFs and clinical notes should be kept in a secure location for 2 years after the last approval of a marketing application or until 2 years have elapsed since formal discontinuation of product development.

8.2 Data management in the immunology laboratories

Standardised operating procedures will be followed in all laboratories to ensure the quality of the data. Data will be stored electronically in an agreed format and datafiles transferred to CTU for the main analysis.

8.3 Data management at the Hospital Clinic CTU

Hospital Clinic CTU will be responsible for:

- Design of the case record forms in collaboration with the Investigators
- The database applications that will contain the computerised trial data
- Data entry for the clinical trial other than the immunologic assays
- Monitoring the trial according to Hospital Clinic GCP guidelines (derived from ICH guidelines) including monitoring vaccine accountability, and dispatch and arrival of immunological specimens
- Preparation of reports to assist the monitoring
- Holding a record for each participant which contains the original top copy of the CRF and documentation detailing all the changes made subsequent to monitoring visits, queries raised and how they were addressed
- Coordination of the committee and group meetings in collaboration with the Investigators
- Coordination of end-point committee meetings, if required, to review the grading of adverse events and their relationship to vaccine product and ELIPSPOT results
- Preparation of analysis files from the database prior to analyses

All CRFs and laboratory reports returned to Hospital Clinic CTU will be reviewed by the Data Management Team, according to Hospital Clinic CTU standardised operating procedures. CRFs will be checked for completeness and passed for review by the trial physician at CTU if required. Data will be coded using the Hospital Clinic system and entered onto a computerised database. Consistency checks and range checks will be performed at the data

entry level.

A printout of the entered data will be generated for each participant CRF in order to cross check the trial database against the CRFs to validate data entry.

The data manager or their deputy will review adverse events, as they arise. Queries raised will be directed to the investigators at Hospital Clínic, Hospital Germans Trias I Pujol or Hospital Gregorio Marañón by letter, fax, email or at a monitoring visit.

Prior to analysis, the safety data will be checked, adverse events validated and data extracted in order for the trial statisticians to run the analysis and prepare the tables.

8.4 Monitoring by HOSPITAL CLÍNIC CTU

Staff from the Hospital Clinic CTU will visit the clinical centres to validate trial data held on the database against the clinical records, and monitor the centre's adherence to GCP and the trial protocol. The Clinical Investigators and participants, by giving consent, agree that the Hospital Clinic CTU may consult and/or copy source records (clinical notes and laboratory values) in order to do this. Such information will be treated as strictly confidential and will in no circumstances be made publicly available. The monitoring will adhere to Hospital Clinic Good Clinical Practice guidelines (derived from ICH guidelines). The following data should be verifiable from source documents:

- documentation of any existing conditions or past conditions relevant to eligibility
- signed consent
- dates of visits including dates of immunisations
- a sample of reported laboratory results
- a description and measurements of cutaneous reactions to immunisation
- grade 3 or 4 adverse events and any events leading to discontinuation of the immunisation schedule
- concomitant prescribed medication

Vaccine returns will also be monitored at visits to the clinical sites.

8.5 Data Ownership

The data generated in this study will be the property the Investigators and will be held on their behalf by Hospital Clinic CTU.

8.6 Trial Coordinating Committee (TCC)

The supervision of the trial will be the responsibility of the Trial Coordinating Committee (TCC). The committee will have a chair, Professor José M Gatell, and the members will include Dr. Felipe García, Dr. Juan Carlos López Bernaldo de Quirós, Dr. Bonaventura Clotet and one further independent member. Additional non-voting members will attend as appropriate to the contents of the meeting. This committee will be responsible for final decisions about grade of adverse events and relationship to study vaccine, and the independent members will have an additional casting vote should this be required. Notes of meetings will be kept.

The trial may be terminated by this Committee for any reason, including on the recommendation of the DSMC.

8.6.1 Trial Management Group (TMG)

This group will oversee the day to day running of the trial and the members will be primarily the clinical and data management teams. The immunologists will join if there are relevant items on the agenda. Notes will be taken and will form the basis of the progress report to the Trial Coordinating Committee.

8.7 Data and Safety Monitoring Committee (DSMC)

A DSMC will be invited to oversee this trial. No member of the Trial Management Group, Trial Coordinating, or any Clinical Investigator responsible for the clinical care of trial participants may be a member of the DSMC.

The DSMC will review the design of the trial prior to commencement. The DSMC will not meet unless there are indications for an interim unblinded review (see below).

8.7.1 Indications for interim review

If the Clinical Investigators report 3 or more participants with any of the following:

- a grade 3 or 4 clinical adverse event confirmed at a visit and not resolved within 72 hours, which is unexpected but considered probably or possibly related to vaccine
- a grade 3 or 4 laboratory event confirmed on repeat testing and not resolved within 72 hours, which is unexpected but considered related or possibly related to vaccine
- an event resulting in a clinical decision to discontinue further immunisations

then the DSMC will be asked to look at these cases unblinded, and trial entry will be suspended.

8.7.2 Indications for discontinuation of immunisations in all participants

If 3 or more of the participants reviewed by the DSMC received MVA B then immunisations will be discontinued pending an unblinded review of all safety data by the DSMC. Following this review the DSMC will make a recommendation to the Trial Coordinating Committee about continuation of the trial.

Unless the trial is discontinued the Trial Coordinating Committee, staff at the Hospital Clinic Clinical Trials Unit (except statisticians who supply confidential analyses to the DSMC) and Clinical Investigators will not be made aware of any unblinded results.

9. Statistical considerations

9.1 Sample size

It is not the remit of this study to recruit a sufficient number of participants to be statistically confident about the result. However by the end of this study at least 20 participants will have been exposed to the study vaccine schedule of interest and this provides the following confidence around observed severe event proportions of 0, 1, 2 and 3 as follows:

Observed event	95% confidence interval if n=24
0	0 – 17%
1	0.1 – 25%
2	1 – 32%
3	3.2 – 38%

This is an exploratory study, and the immunological analyses will be descriptive.

9.2 Analysis

All safety end-points will be graded by the Clinical Investigators and reviewed by the Trial Management Group. Any queries about grade and relationship to study product that cannot be resolved will be referred to the Trial Coordinating Committee for a final decision. This will be undertaken blinded.

All clinical event and routine laboratory data will be included in the safety analysis. Tables including all events occurring within 28 days of an immunisation, and limited to those possibly or probably related to study product will be prepared.

The immunological end-points will be considered as change from baseline value for the main analysis. There will be an immunological end-point committee, which will undertake a blinded review of the ELISPOT assays and attempt to quantify each result.

Figures/tables will be prepared including all participants' results, and limited to those participants who completed the full schedule.

10. Confidentiality, ethics and responsibilities, including indemnity

Full medical confidentiality will be preserved.

The study will be conducted according to the Hospital Clinic GCP guidelines (based on ICH guidelines) and the Declaration of Helsinki (version 1996), and it is the responsibility of the Clinical Investigators and the staff at the Hospital Clinic CTU to abide by this protocol.

The Principal and Clinical Investigators are responsible for obtaining the appropriate Local Research Ethics Committee (LREC) approval for the study protocol, the subject information sheet and the consent form. An ethical approval letter stating the title, protocol number. The Principal and Clinical Investigators are responsible for informing the LRECs of any SAEs as required, and submitting annual reports as required.

Regulatory submissions will be made in Spain. Regulatory approval must be provided before study materials will be shipped to the clinical centres.

CTU will be responsible for preparing the randomisation list, all aspects of data management including monitoring of the clinical sites, and the analysis. Staff will also be responsible for coordinating the response to any SAEs that arise during the course of the trial and reporting these if indicated to the regulatory authorities.

Hospital Clinic will act as Sponsor for the trial, and will coordinate the necessary clinical trial agreement delineating the above responsibilities and the liability for events occurring as a result of participating on the trial. This agreement must be signed by all parties involved before materials are shipped to the relevant clinical centre. Indemnity will be ensured by the following parties:

- The Principal Investigators will ensure that all clinical staff engaged in the study are covered for negligent harm to a participant, either through a personal or hospital insurance scheme
- Infectious Diseases Unit, Hospital Clinic will be responsible for ensuring that a policy is in place to cover 'no fault compensation' for participants on the trial who suffer an adverse event that is attributable to participation in the trial but which is not related to the manufacturing fault of the product or due to negligent harm caused by the clinical staff.

11 Publication

It is intended that the results of this study will be published in an appropriate peer-reviewed journal, with the aim of submitting a paper for publication within 6 months of the study's completion. No other publications, whether in writing or verbally, will be made before the definitive manuscript has been agreed and accepted for publication, without the prior approval of this committee.
